# PIM1 is a potential therapeutic target for the leukemogenic effects mediated by JAK/STAT pathway mutations in T-ALL/LBL

**DOI:** 10.1038/s41698-024-00638-2

**Published:** 2024-07-20

**Authors:** Antonio Lahera, Laura Vela-Martín, Pablo Fernández-Navarro, Pilar Llamas, José L. López-Lorenzo, Javier Cornago, Javier Santos, José Fernández-Piqueras, María Villa-Morales

**Affiliations:** 1 Department of Biology, Universidad Autónoma de Madrid, Madrid, 28049 Spain; 2 Department of Genome dynamics and function, Centro de Biología Molecular Severo Ochoa (CBM), Consejo Superior de Investigaciones Científicas-Universidad Autónoma de Madrid (CSIC-UAM), Madrid, 28049 Spain; 3 Area of Genetics and Genomics, IIS Fundación Jiménez Díaz, Madrid, 28040 Spain; 4 Unit of Cancer and Environmental Epidemiology, Centro Nacional de Epidemiología, Instituto de Salud Carlos III, Madrid, 28029 Spain; 5 Consorcio de Investigación Biomédica de Epidemiología y Salud Pública (CIBERESP), Madrid, 28029 Spain; 6 Division of Hematology and Hemotherapy, Hospital Universitario Fundación Jiménez Díaz, Madrid, 28040 Spain; 7 Institute for Molecular Biology-IUBM (Universidad Autónoma de Madrid) Madrid, Madrid, 28049 Spain

**Keywords:** Acute lymphocytic leukaemia, Targeted therapies, Molecular medicine

## Abstract

Precursor T-cell neoplasms (T-ALL/LBL) are aggressive hematological malignancies that arise from the malignant transformation of immature thymocytes. Despite the JAK/STAT pathway is recurrently altered in these neoplasms, there are not pharmacological inhibitors officially approved for the treatment of T-ALL/LBL patients that present oncogenic JAK/STAT pathway mutations. In the effort to identify potential therapeutic targets for those patients, we followed an alternative approach and focused on their transcriptional profile. We combined the analysis of molecular data from T-ALL/LBL patients with the generation of hematopoietic cellular models to reveal that JAK/STAT pathway mutations are associated with an aberrant transcriptional profile. Specifically, we demonstrate that JAK/STAT pathway mutations induce the overexpression of the *PIM1* gene. Moreover, we show that the pan-PIM inhibitor, PIM447, significantly reduces the leukemogenesis, as well as the aberrant activation of c-MYC and mTOR pathways in cells expressing different JAK/STAT pathway mutations, becoming a potential therapeutic opportunity for a relevant subset of T-ALL/LBL patients.

## Introduction

Precursor lymphoid neoplasms are aggressive hematological malignancies that represent the most common type of cancer among children and are associated with poor outcome in adult patients^1^. Such hematological malignancies can be divided according to the cell of origin into neoplasms compromised with the B-cell or the T-cell lineage. Specifically, precursor T-cell neoplasms arise from the malignant transformation of immature thymocytes at various differentiation stages^2^. They often affect the bone marrow and blood (Acute T-cell lymphoblastic leukemia, T-ALL) though less frequently they appear as mass lesions in the thymus or lymph nodes (T-cell lymphoblastic lymphoma, T-LBL)^3^.

Current standard-of-care treatments for T-ALL/LBL mainly consist of high-dose multi-agent chemotherapy followed by hematopoietic stem cell transplantation (HSCT) in standard-high risk patients^4,5^. Despite these treatments achieve reasonable rates of initial complete responses (CR), they are associated with acute and long-term toxicity. In addition, those patients who relapse or do not respond to first-line treatments show a dismal prognosis with survival rates below 10% and very limited therapeutic options^6^. Therefore, there is an urgent need to implement novel personalized therapies with greater efficacy and fewer adverse effects against the signaling pathways that are recurrently deregulated in T-ALL/LBL^7^.

The JAK/STAT pathway is the second most frequently altered signaling pathway in T-ALL/LBL only behind the NOTCH1 pathway^8–10^. Consequently, about 25% of T-ALL/LBL patients show oncogenic mutations in different members of the JAK and STAT gene families that promote constitutive activation of the JAK/STAT pathway and sustained phosphorylation of STAT5, leading to tumor development^11–17^. Unfortunately, there is no specific treatment for T-ALL/LBL patients with JAK/STAT pathway mutations since they can affect several genes and usually appear simultaneously. Therefore, multiple pharmacological inhibitors as well as their combinations would be needed, complicating the implementation of novel personalized therapies^18,19^. Moreover, due to issues with selectivity and poor druggability, the inhibition of STAT proteins results challenging and no pharmacological inhibitors have been approved against them^7^.

In the present manuscript, we followed an alternative approach and focused on the transcriptional profile of T-ALL patients with JAK/STAT pathway mutations since, once phosphorylated, STAT proteins become active and translocate to the nucleus, where they act as transcription factors and promote the expression of several genes that participate in different cellular processes^20^. Therefore, those genes involved in oncogenesis and upregulated as a consequence of JAK/STAT pathway mutations may become potential therapeutic targets for the development of novel personalized therapies against a relevant fraction of T-ALL cases.

## Results

### JAK/STAT pathway mutations are associated with an aberrant transcriptional profile

**Fig. 1:**
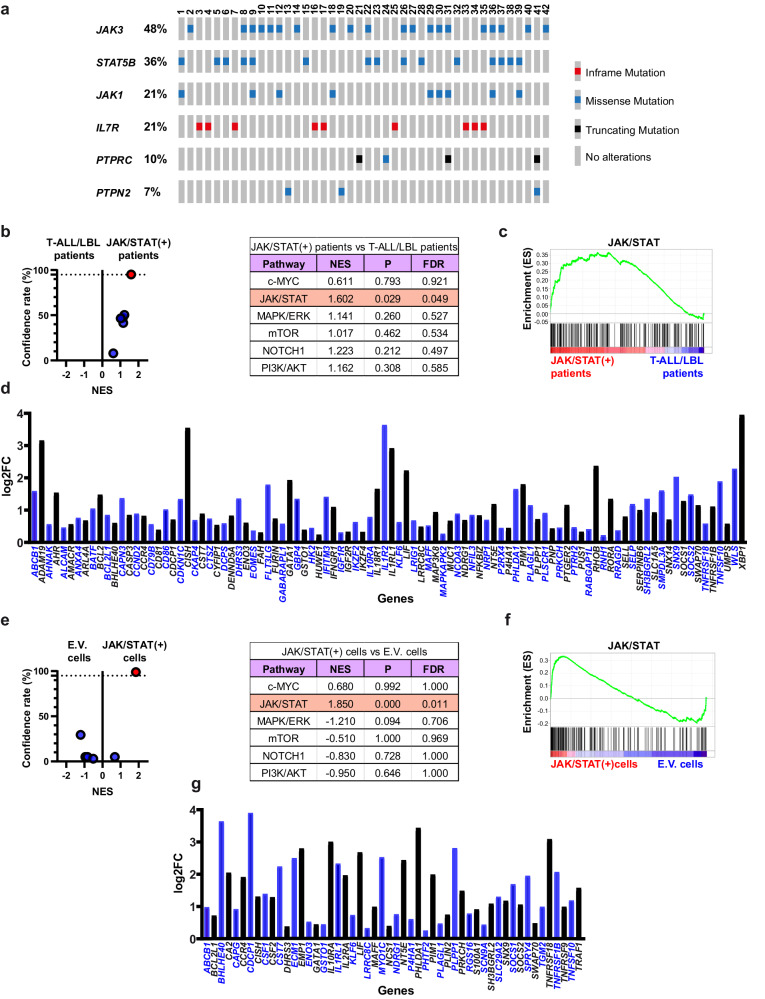
JAK/STAT pathway mutations are associated with an aberrant transcriptional profile. **a** Representative scheme of mutations susceptible of contributing to the constitutive activation of the JAK/STAT pathway in T-ALL/LBL patients. Rows correspond to the selected genes, which are ordered according to their mutation rate in the cohort. Columns represent the tumor samples for which alterations were detected (*n* = 42). Figure generated with the Oncoprint tool in cBioPortal (https://www.cbioportal.org/oncoprinter)^63,64^. **b** Volcano plot showing the results for multiple GSEA-signatures between patients with JAK/STAT pathway mutations (referred as JAK/STAT (+) patients) and the rest of T-ALL/LBL patients (referred as T-ALL/LBL patients). The selected GSEA-signatures correspond to different signaling pathways that are recurrently deregulated in T-ALL/LBL and whose deregulation may promote tumor development. Those signatures with a confidence rate above 95% are highlighted in red. Data derived from the analysis are shown in the adjacent chart: NES, normalized enrichment score; P, nominal *p* value; FDR, false discovery rate. **c** GSEA-plot for the JAK/STAT pathway in patients with and without JAK/STAT pathway mutations. **d** RNA-seq expression of the leading-edge genes from the JAK/STAT pathway signature that are significantly upregulated in patients with JAK/STAT-pathway mutations compared to the rest of T-ALL/LBL patients. **e** Volcano plot showing the results for multiple GSEA-signatures between M07e cells transduced with JAK3^Q988P^ (referred as JAK/STAT (+) cells) and M07e cells transduced with an empty vector (referred as E.V. cells). The selected GSEA-signatures correspond to different signaling pathways that are recurrently deregulated in T-ALL/LBL and whose deregulation may promote tumor development. Those signatures with a confidence rate above 95% are highlighted in red. Data derived from the analysis are shown in the adjacent chart. **f** GSEA-plot for the JAK/STAT pathway in cells with and without a JAK/STAT pathway mutation. **g** RNA-seq expression of the leading-edge genes from the JAK/STAT pathway signature that are significantly upregulated in M07e cells transduced with JAK3^Q988P^ compared to M07e cells transduced with an empty vector.

We first examined whether T-ALL/LBL patients with JAK/STAT pathway mutations show an aberrant transcriptional profile that is associated with an increased JAK/STAT pathway activation. To this end, we analyzed exome data from patients diagnosed with T-ALL/LBL and classified them according to the presence or absence of JAK/STAT pathway mutations. We observed JAK/STAT pathway mutations in more than 15% of the patients (*n* = 42) (Supplementary Table 1). Specifically, we identified a total of 67 mutations affecting 6 different JAK/STAT pathway members whose alteration is susceptible to promote constitutive activation of the JAK/STAT pathway and sustained phosphorylation of STAT5^8–10^ (Fig. 1a, Supplementary Table 2). We compared the transcriptional profile of patients with and without JAK/STAT pathway mutations and, specifically, we evaluated the activation status of several signaling pathways that are recurrently deregulated in T-ALL/LBL and whose deregulation may contribute to tumor development (Fig. 1b). In this respect, only the GSEA signature for the JAK/STAT pathway showed a confidence rate above 95% and was significantly enriched in patients with JAK/STAT pathway mutations compared to the rest of T-ALL/LBL patients (Fig. 1c). Subsequently, we accounted for the genes that are associated with an increased JAK/STAT pathway activation and specifically upregulated in patients with JAK/STAT pathway mutations (Fig. 1d).

Next, we experimentally validated that the relationship observed in T-ALL/LBL patients between the upregulation of multiple genes and the presence of JAK/STAT pathway mutations truly derives from the ability of these mutations to induce an aberrant transcriptional profile. For such purpose, we employed complementary hematopoietic cellular models transduced with either a JAK/STAT pathway mutation, specifically JAK3^Q988P^, or an empty vector. Again, only the GSEA signature for the JAK/STAT pathway showed a confidence rate above 95% and was significantly enriched in cells transduced with JAK3^Q988P^ compared to cells transduced with the empty vector (Fig. 1e, f), recapitulating the results previously obtained from the comparison between patients with JAK/STAT pathway mutations and the rest of T-ALL/LBL patients. Subsequently, we accounted for the genes that are associated with an increased JAK/STAT pathway activation and specifically upregulated in cells transduced with the JAK3^Q988P^ mutation (Fig. 1g).

### JAK/STAT pathway mutations induce the upregulation of PIM1 at mRNA and protein levels

**Fig. 2:**
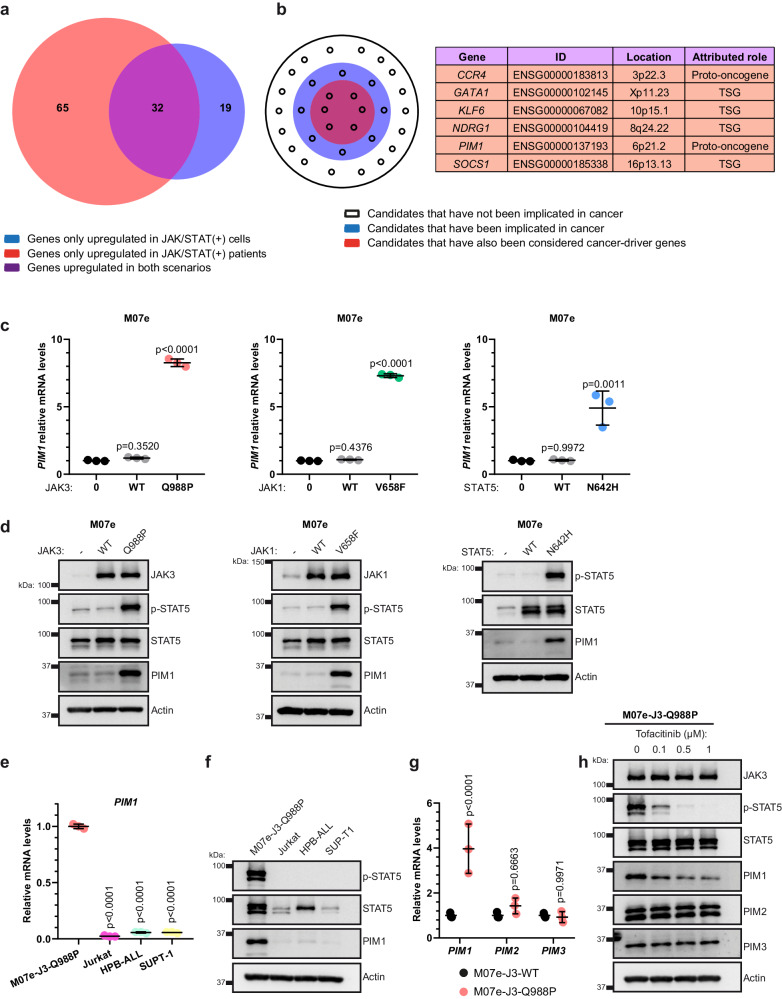
JAK/STAT pathway mutations induce the upregulation of PIM1 at mRNA and protein levels. **a** Venn diagram accounting for the leading-edge genes from the JAK/STAT pathway signature that are significantly upregulated. Genes exclusively upregulated in patients with JAK/STAT pathway mutations (referred as JAK/STAT (+) patients) are marked in red while genes exclusively upregulated in M07e cells transduced with JAK3^Q988P^ (referred as JAK/STAT (+) cells) are marked in blue. Those genes upregulated in both scenarios appear in purple and constitute the strongest candidates to be overexpressed due the aberrant activity of JAK/STAT pathway mutations. **b** Genes upregulated in patients with JAK/STAT pathway mutations as well as in M07e cells transduced with JAK3^Q988P^ were classified regarding their potential to be involved in leukemogenesis. Those candidates that have been implicated in cancer according to the Atlas of Genetics and Cytogenetics in Oncology and Haematology (AGCOH) are marked in blue. Those candidates that have also been considered cancer-driver genes according to IntOgen are marked in red. Genes with the highest probability of being involved in leukemogenesis are summarized in the adjacent chart, indicating: their Ensemble ID, their chromosome location and whether they are cataloged as proto-oncogenes or tumor suppressor genes (TSG). **c** mRNA expression levels by RT-qPCR of *PIM1* in M07e cells transduced with JAK3^Q988P^, JAK1^V658F^ or STAT5^N642H^ compared to cells transduced with the corresponding wild-type sequence and non-transduced cells (0). Statistical comparisons are made against non-transduced cells (0). **d** Western blot for JAK1, JAK3, p-STAT5, STAT5 and PIM1 in M07e cells transduced with JAK3^Q988P^, JAK1^V658F^ or STAT5^N642H^ compared to cells transduced with the corresponding wild-type sequence and non-transduced cells (-). **e** mRNA expression levels by RT-qPCR of *PIM1* in Jurkat, HPB-ALL, SUPT-1 and M07e cells transduced with JAK3^Q988P^. Statistical comparisons are made against M07e-J3-Q988P cells. **f** Western blot for p-STAT5, STAT5 and PIM1 in Jurkat, HPB-ALL, SUPT-1 and M07e cells transduced with JAK3^Q988P^. **g** mRNA expression levels by RT-qPCR of *PIM1*, *PIM2* and *PIM3* in M07e cells transduced with JAK3^WT^ or JAK3^Q988P^. Statistical comparisons are made between M07e-JAK3WT and M07e-J3-Q988P cells. **h** Western blot for JAK3, p-STAT5, STAT5, PIM1, PIM2 and PIM3 in M07e cells transduced with JAK3^Q988P^ untreated or treated with tofacitinib (0.1 μM, 0.5 μM or 1 μM). The graphics show the mean ± standard deviation (s.d.) after three independent experiments. All images are representative examples of at least three independent experiments.

Those genes upregulated in both scenarios, that is, in patients with JAK/STAT pathway mutations and in the hematopoietic cellular model expressing a JAK/STAT pathway mutation, emerged as the strongest candidates to be overexpressed because of the aberrant activity exhibited by JAK/STAT pathway mutations (Fig. 2a). To determine which of the selected genes are likely to promote tumor development, we assessed whether they have been implicated in cancer and whether they have been considered cancer-driver genes (Fig. 2b). Although we identified five genes fulfilling both criteria, only two of them are catalogued as proto-oncogenes and, accordingly, are likely to promote tumor development due to their upregulation. Among them, we specifically focused on *PIM1*, since it codes for a serine/threonine kinase protein that lacks negative regulation at the structural level^21^. Therefore, the catalytic activity of PIM1 is directly related to its expression levels^22^ and PIM1 overexpression may constitute a significant contribution to tumor development in cells with JAK/STAT pathway mutations. Once PIM1 was selected as a promising candidate for further studies, we verified the ability of the JAK3^Q988P^ mutation to induce *PIM1* overexpression in different T-ALL/LBL cell lines (Supplementary Fig. 1a).

To experimentally determine whether *PIM1* overexpression contributes to the leukemogenic effects mediated by JAK/STAT pathway mutations, we generated hematopoietic cellular models that rely on different JAK/STAT pathway mutations for their growth and viability. Specifically, we focused on mutations affecting the *JAK1, JAK3* and *STAT5* genes, since these are the most frequently altered members of the JAK/STAT pathway in the patient cohort that we have analyzed (Supplementary Fig. 1b, c). Cells transduced with the selected mutations showed significantly higher levels of *PIM1* expression than untransduced cells or cells transduced with the wild-type sequences of the corresponding genes (Fig. 2c, Supplementary Fig. 2a). Furthermore, we observed that PIM1 upregulation not only occurs at the mRNA level but also at the protein level in both hematopoietic cellular models (Fig. 2d, Supplementary Fig. 2b). We corroborated the absence of PIM1 in multiple T-ALL/LBL-derived cell lines lacking JAK/STAT pathway mutations (Fig. 2e, f), further supporting the relationship between these mutations and PIM1 upregulation. Since *PIM1* belongs to a gene family composed of three members (*PIM1, PIM2* and *PIM3*), we also evaluated whether *PIM1* is the main member of the PIM family whose expression is upregulated due to the aberrant activity exhibited by JAK/STAT pathway mutations. Cells transduced with JAK3^Q988P^ showed a significant *PIM1* overexpression compared to cells transduced with JAK3^WT^, while the expression levels for *PIM2* and *PIM3* remained similar in both cell types (Fig. 2g). Furthermore, in cells transduced with JAK3^Q988P^, the treatment with the JAK3 inhibitor tofacitinib reversed PIM1 upregulation without affecting the protein levels for PIM2 and PIM3 (Fig. 2h).

### PIM447 reduces the proliferation, viability and G1/S transition of cells with JAK/STAT pathway mutations

**Fig. 3:**
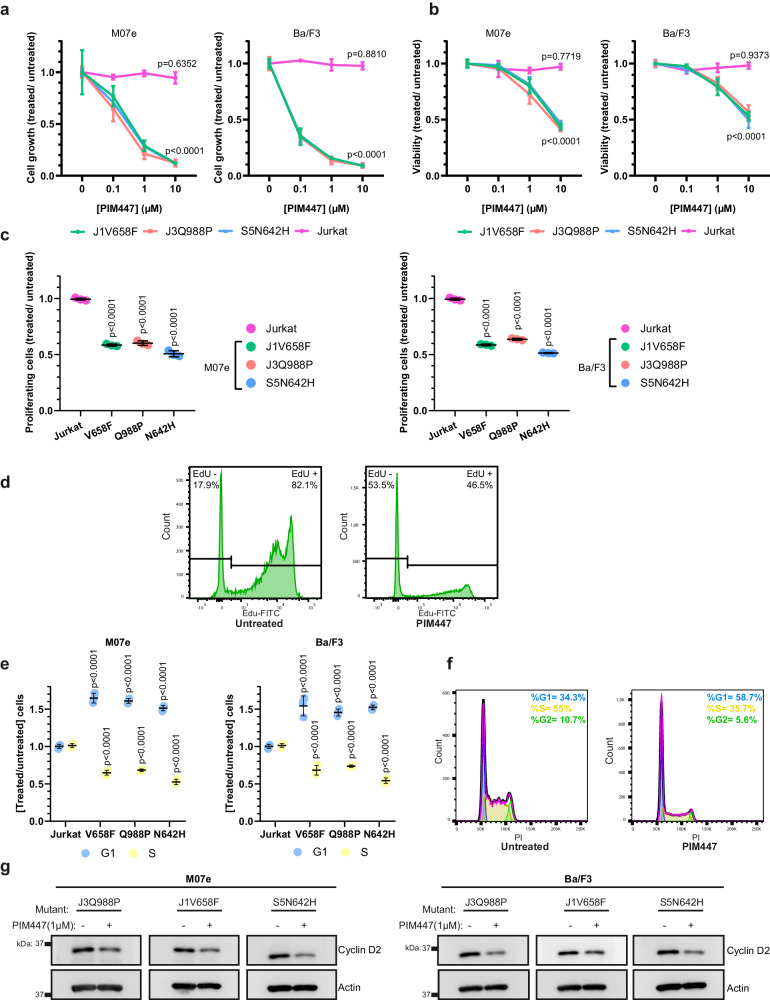
PIM447 reduces the proliferation, viability and G1/S transition of cells with JAK/STAT pathway mutations. Cell growth (**a**) and viability (**b**) assays of M07e (left) and Ba/F3 cells (right) transduced with JAK3^Q988P^, JAK1^V658F^ or STAT5^N642H^, treated with PIM447 (0.1 μM, 1 μM or 10 μM) and referred to untreated cells. Jurkat cells were used as a negative control since they lack oncogenic JAK/STAT pathway mutations and *PIM1* overexpression. Statistical comparisons are made against untreated cells. **c** Cell proliferation analysis of M07e (left) and Ba/F3 cells (right) transduced with JAK3^Q988P^, JAK1^V658F^ or STAT5^N642H^, treated with PIM447 (1 μM) and referred to untreated cells. Statistical comparisons are made against Jurkat cells. **d** Representative images for M07e cells transduced with JAK3^Q988P^ are depicted. **e** Cell cycle analysis of M07e (left) and Ba/F3 cells (right) transduced with JAK3^Q988P^, JAK1^V658F^ or STAT5^N642H^, treated with PIM447 (1 μM) and referred to untreated cells. Statistical comparisons are made against Jurkat cells. **f** Representative images for M07e cells transduced with JAK3^Q988P^ are depicted. **g** Western blot for CyclinD2 in M07e (left) and Ba/F3 cells (right) transduced with JAK3^Q988P^, JAK1^V658F^ or STAT5^N642H^ and treated with PIM447 (1 μM) or left untreated. The graphics show the mean ± standard deviation (s.d.) after three independent experiments. All images are representative examples of at least three independent experiments.

Our results show that different JAK/STAT pathway mutations induce *PIM1* overexpression, postulating PIM1 activity as a relevant event for the leukemogenic effects mediated by the JAK/STAT pathway mutations identified in T-ALL/LBL, as well as a potential therapeutic target in this malignancy. Consequently, we evaluated the effects of the pan-PIM inhibitor PIM447 on the different cellular models that we have generated and that rely on JAK/STAT pathway mutations for their growth and viability. Our results demonstrate that PIM447 significantly reduces the growth of such cellular models but not of Jurkat cells (Fig. 3a), which serve as a negative control, since they lack JAK/STAT pathway mutations and PIM1 upregulation. To determine whether the observed reduction in cell growth is the consequence of a decrease in cell division, an increase in cell death or both, we analyzed proliferation and viability rates. Our results demonstrate that PIM447 significantly reduces the levels of viability (Fig. 3b) and proliferation (Fig. 3c, d) in cells with JAK/STAT pathway mutations. To assess whether the reduction observed in proliferation is associated with a cell cycle blockage, we analyzed the cell cycle profile and observed that PIM447 significantly reduces the G1/S transition (Fig. 3e, f) and the levels of cyclinD2 (Fig. 3g), emerging as a potential treatment against the leukemogenic effects mediated by JAK/STAT-pathway mutations.

### PIM447 inhibits the aberrant activation of c-MYC and mTOR pathways in cells relying on different JAK/STAT pathway mutations for leukemogenesis

**Fig. 4:**
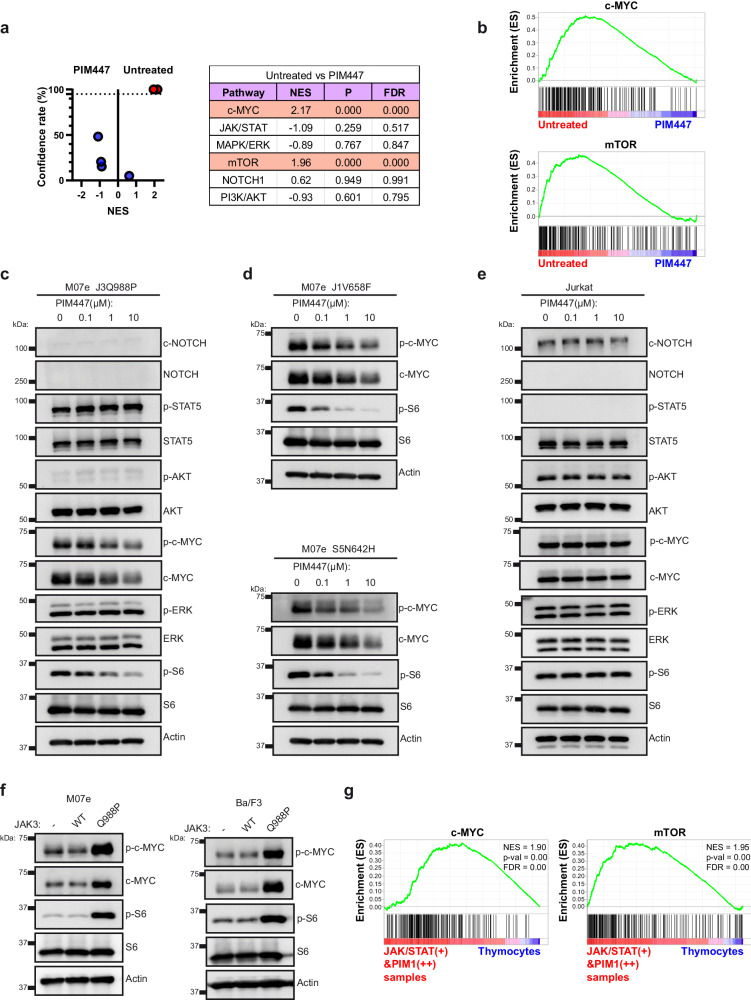
PIM447 inhibits the aberrant activation of c-MYC and mTOR pathways in cells relying on different JAK/STAT pathway mutations for leukemogenesis. **a** Volcano plot showing the results for multiple GSEA-signatures between M07e-JAK3^Q988P^ cells untreated (referred as untreated) and M07e-JAK3^Q988P^ cells treated with PIM447 for 24 hours (referred as PIM447). The selected GSEA-signatures correspond to different signaling pathways that are recurrently deregulated in T-ALL/LBL and whose deregulation may promote tumor development. Those signatures with a confidence rate above 95% are highlighted in red. Data derived from the analysis are shown in the adjacent chart: NES, normalized enrichment score; P, nominal *p* value; FDR, false discovery rate. **b** GSEA-plots for c-MYC and mTOR pathways in M07e-JAK3^Q988P^ cells untreated and treated with PIM447. **c** Western blot for c-NOTCH1, NOTCH1, p-STAT5, STAT5, p-AKT, AKT, p-c-MYC, c-MYC, p-ERK, ERK, p-S6 and S6 in M07e cells transduced with JAK3^Q988P^ untreated or treated with PIM447 (0.1 μM, 1 μM or 10 μM). **d** Western blot for p-c-MYC, c-MYC, p-S6 and S6 in M07e cells transduced with JAK1^V658F^ or STAT5^N642H^ untreated or treated with PIM447 (0.1 μM, 1 μM or 10 μM). **e** Western blot for c-NOTCH1, NOTCH1, p-STAT5, STAT5, p-AKT, AKT, p-c-MYC, c-MYC, p-ERK, ERK, p-S6 and S6 in Jurkat cells untreated or treated with PIM447 (0.1 μM, 1 μM or 10 μM). **f** Western blot for p-c-MYC, c-MYC, p-S6 and S6 in M07e (left) and Ba/F3 cells (right) untransduced (-) or transduced with *JAK3*^WT^ or JAK3^Q988P^. **g** GSEA-plots for c-MYC and mTOR pathways in control thymocytes (referred as thymocytes) and T-ALL/LBL samples with JAK/STAT pathway mutations and a twofold increase in *PIM1* expression (referred as JAK/STAT (+) &PIM1(++) samples). We selected human postnatal thymocytes as the most suitable control since T-ALL/LBL samples without mutations in the JAK/STAT pathway may present other molecular alterations that are susceptible of increasing the GSEA-signatures analyzed. All images are representative examples of at least three independent experiments.

PIM447 significantly reduces the proliferation, viability and G1/S transition of cells with JAK/STAT pathway mutations, suggesting that the activity of PIM proteins promotes an aberrant activation of the molecular cascades that are essential for the leukemogenic effects mediated by JAK/STAT pathway mutations. To test such hypothesis, we analyzed transcriptomic data from cells expressing JAK3^Q988P^ that were treated with PIM447 or left untreated. We performed GSEA analysis and evaluated the activation status of several signaling pathways that are recurrently deregulated in T-ALL and whose deregulation contributes to tumor development (Fig. 4a). In this respect, only the GSEA signatures for c-MYC and mTOR pathways showed a confidence rate above 95% and were significantly enriched in untreated cells compared to PIM447-treated cells (Fig. 4b). We next confirmed that PIM447 reduces the activation of c-MYC and mTOR pathways by Western Blot. PIM447-treated cells showed reduced phosphorylation levels for S6, an essential member of the mTOR pathway, and c-MYC, while no differences were observed for proteins belonging to other signaling pathways (Fig. 4c, Supplementary Fig. 3a). We also confirmed reduced phosphorylation levels for c-MYC and S6 in cells expressing JAK1^V658F^ or STAT5^N642H^ after PIM447 treatment (Fig. 4d, Supplementary Fig. 3b). Notably, PIM447 had no effect on c-MYC and S6 phosphorylation in Jurkat cells (Fig. 4e). Furthermore, cells transduced with the JAK3^Q988P^ mutation showed higher phosphorylation levels for c-MYC and S6 than untransduced cells or cells transduced with the wild-type sequence of *JAK3* (Fig. 4f). In the clinical context, the GSEA signatures for c-MYC and mTOR pathways were significantly enriched in T-ALL/LBL samples with JAK/STAT pathway mutations and elevated *PIM1* expression compared to control thymocytes (Fig. 4g). Therefore, our results show that JAK/STAT pathway mutations lead to the aberrant activation of c-MYC and mTOR pathways and that such activation can be reversed by PIM447 treatment.

### Inhibition of PIM proteins leads to changes in gene expression patterns

**Fig. 5:**
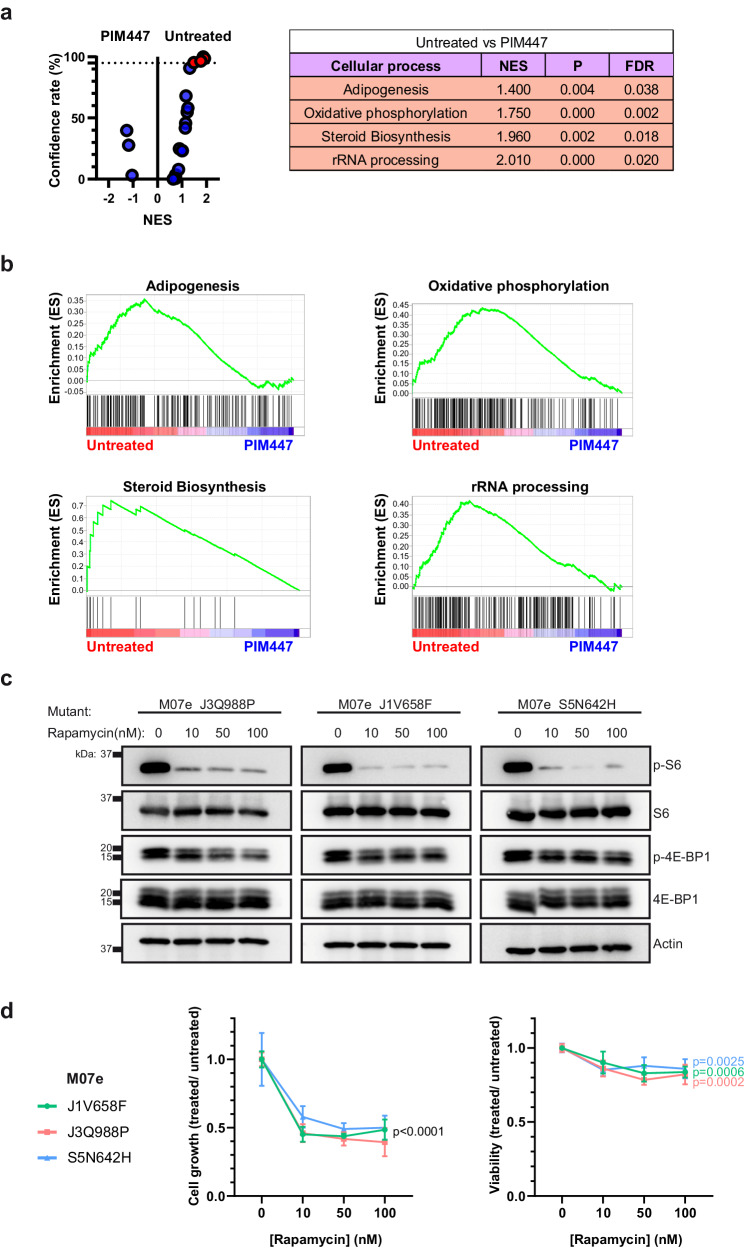
Inhibition of PIM proteins leads to changes in gene expression patterns. **a** Volcano plot showing the results for multiple GSEA-signatures between M07e-JAK3^Q988P^ cells untreated (referred as untreated) and M07e-JAK3^Q988P^ cells treated with PIM447 for 24 hours (referred as PIM447). The selected GSEA-signatures correspond to different cellular processes that have been implicated in cancer. Those signatures with a confidence rate above 95% are highlighted in red. Data derived from the analysis of the significantly enriched signatures are shown in the adjacent chart: NES, normalized enrichment score; P, nominal *p* value; FDR, false discovery rate. **b** GSEA-plots for adipogenesis, oxidative phosphorylation, steroid biosynthesis and rRNA processing in M07e-JAK3^Q988P^ cells untreated and treated with PIM447. **c** Western blot for p-S6, S6, p-4E-BP1 and 4E-BP1 in M07e cells transduced with JAK3^Q988P^, JAK1^V658F^ or STAT5^N642H^ and treated with rapamycin (10 nM, 50 nM or 100 nM) or left untreated. **d** Cell growth (left) and viability (right) assays of M07e cells transduced with JAK3^Q988P^, JAK1^V658F^ or STAT5^N642H^, treated with rapamycin (10 nM, 50 nM or 100 nM) and referred to untreated cells. Statistical comparisons are made against untreated cells. The graphics show the mean ± standard deviation (s.d.) after three independent experiments. All images are representative examples of at least three independent experiments.

Notably, c-MYC and mTOR pathways participate in the transcription of multiple genes, postulating the activity of PIM proteins in cells with JAK/STAT pathway mutations as a necessary event for the mRNA expression of gene sets involved in different cellular processes. Therefore, we evaluated through GSEA the mRNA expression levels for several gene sets corresponding to cellular processes that have been reported to play a significant role in tumor development (Fig. 5a, Supplementary Table 3). Among them, only the GSEA signatures for adipogenesis, oxidative phosphorylation, steroid biosynthesis and rRNA processing showed a confidence rate above 95% and were significantly enriched in untreated cells compared to PIM447-treated cells (Fig. 5b). We next confirmed the results in the clinical context through the comparison between control thymocytes and T-ALL samples with JAK/STAT pathway mutations and elevated PIM1 expression (Supplementary Fig. 4a), further supporting the relevance of PIM1 activity for the mRNA expression of specific gene sets. Finally, c-MYC and mTOR pathways have been implicated in oncogenesis, so we wondered whether their pharmacological inhibition could be effective against the leukemogenic effects mediated by JAK/STAT pathway mutations. In this regard, c-MYC is a transcription factor whose pharmacological inhibition is complicated whereas mTOR codes for a serine/threonine kinase that can be specifically inhibited by different pharmacological compounds. Therefore, we analyzed the effects of the mTOR inhibitor rapamycin on the different hematopoietic cellular models that we have previously generated and that rely on JAK/STAT pathway mutations for their growth and viability. Our results demonstrate that rapamycin efficiently inhibited the mTOR pathway by reducing the phosphorylation levels of S6 and 4EBP1 (Fig. 5c, Supplementary Fig. 4b). However, even at the highest recommended dose for rapamycin, the effects on cell growth and viability are only partial when compared to those previously observed for PIM447 (Fig. 5d, Supplementary Fig. 4c), indicating that the activity of PIM proteins supports tumor development through additional mechanisms than simply activating the mTOR signaling pathway and postulating PIM447 as a more suitable treatment in terms of efficacy.

## Discussion

The molecular basis underlying precursor T-cell neoplasms are highly heterogeneous and may involve deregulation of multiple signaling pathways such as NOTCH1, JAK/STAT, PI3K/AKT, MAPK/ERK, c-MYC or mTOR^8–10^. This scenario complicates the development of personalized therapies and since the FDA approval of nelarabine in 2005 no new treatments have been implemented for T-ALL/LBL patients^23–25^.Therefore, it is essential to identify potential therapeutic targets for the development of personalized therapies against the most frequently deregulated signaling pathways in precursor T-cell neoplasms. While gamma-secretase and PSEN1 inhibitors are promising candidates for the treatment of T-ALL/LBL patients with NOTCH1 pathway mutations^26–29^, pharmacological inhibition of the JAK/STAT pathway is significantly more complex due to the presence of oncogenic mutations in multiple pathway members as well as specificity and selectivity issues affecting inhibitors against STAT proteins^8–10^. In consequence, despite the JAK/STAT pathway is the second most frequently deregulated signaling pathway in precursor T-cell neoplasms, there is no specific treatment for T-ALL/LBL patients with JAK/STAT pathway mutations^7^.

To identify aberrantly expressed genes in T-ALL/LBL patients with JAK/STAT pathway mutations, we compared their transcriptional profile with that of patients without mutations in the JAK/STAT-pathway. Our results showed that JAK/STAT pathway mutations are associated with an aberrant transcriptional profile involving the upregulation of multiple genes. Among the different candidates, we focused on those previously associated with cancer and specifically on *PIM1*, which encodes a serine/threonine-kinase protein, since protein phosphorylation is essential for signal transduction^30^ and aberrant phosphorylation is tightly associated with tumor development^31^. Moreover, PIM proteins are characterized for being in a constitutively active conformation and not presenting any kind of negative regulation at the structural level, so their catalytic activity mostly depends on their expression levels and thus, *PIM1* overexpression might represent a direct contribution to tumor development^21,22^. In this regard, we not only observed a relationship between *PIM1* mRNA upregulation and the presence of JAK/STAT pathway mutations, in agreement with a previous report from a different patient cohort^32^, but also employed complementary cellular models to demonstrate that such correlation truly derives from the ability of JAK/STAT pathway mutations to induce *PIM1* overexpression. Furthermore, we demonstrated that *PIM1* upregulation occurs at both mRNA and protein levels and thus, is susceptible to have an impact on the phosphorylation rates. In addition, we confirmed that *PIM1* is the main member of the *PIM* family whose expression levels are modulated by JAK/STAT-pathway oncogenic mutations, indicating a specific association between such mutations and *PIM1*.

We then investigated whether PIM1 inhibition could become an effective treatment against those cells that rely on JAK/STAT pathway mutations for their growth and viability. However, individualized inhibition of a single PIM family member is complicated due to specificity and overcompensation issues, which may cause aberrant up-regulation of the remaining members, yielding only limited effect, especially given that PIM1, PIM2 and PIM3 exhibit a remarkable degree of functional redundancy^33,34^. Therefore, simultaneously inhibiting the catalytic activity of the three PIM family members through the treatment with pan-PIM inhibitors is considered the most reliable therapeutic approach in the clinical context^35–38^. Moreover, pan-PIM inhibitors have been reported as highly specific, since PIM proteins exhibit a remarkable level of homology and are the only kinases with a proline at the hinge of the active center, resulting in a single hydrogen bridge interacting with the ATP molecule^39,40^. Notably, the triple knockout of *PIM1*, *PIM2*, and *PIM3* results in viable and fertile mice, so the simultaneous inhibition of the three PIM family members would be exempt from severe adverse side effects^41^.

Given the relevance of PIM proteins, previous studies have evaluated the efficacy of pan-PIM inhibitors in T-ALL/LBL but the reported results are heterogeneous, with some cellular models showing high sensitivity to the inhibition of PIM proteins while others are fully resistant^33,42–44^. Therefore, it is essential to reveal the underlying molecular basis for efficiently discriminating between sensitive and resistant cellular models in order to identify those T-ALL/LBL neoplasms that may become potential candidates for treatment with pan-PIM inhibitors. The first evidence came with translocations involving the *PIM1* gene^42^. However, such alterations are extremely rare and have a frequency below 1% so pan-PIM inhibitors would only be useful for a small number of T-ALL/LBL patients^8^. Another example would be IL-7 responsiveness, since some of the T-ALL/LBL neoplasms that respond to IL-7 are also sensitive to the inhibition of PIM proteins^43^. However, IL-7 responsiveness is present in a limited fraction of T-ALL neoplasms, and of these, only a minority show mRNA expression levels of *PIM1* that are high enough to be considered a potential therapeutic target^44^. Moreover, identifying those tumors that respond to IL-7, as well as the magnitude of the response, is a difficult exercise that complicates its implementation during the initial diagnosis of the patient and the molecular analysis of the tumor^6^. Notably, although JAK/STAT pathway mutations have been reported in about 30% of T-ALL patients^8,10^, none of the previous studies evaluating the efficacy of pan-PIM inhibitors in T-ALL have studied cellular models with JAK/STAT pathway mutations. Therefore, this manuscript provides substantial evidence that a pan-PIM inhibitor significantly reduces the leukemogenic effects mediated by JAK/STAT pathway mutations identified in T-ALL. Specifically, our results demonstrate that the recently developed pan-PIM inhibitor PIM447^37,38^ impairs the proliferation, viability and G1/S transition of cells with JAK/STAT pathway mutations postulating PIM447 as a potential therapeutic option against tumor cells bearing these alterations.

Next, we studied the subjacent molecular cascades and revealed that JAK/STAT pathway mutations identified in T-ALL lead to the aberrant activation of c-MYC and mTOR pathways and that such activation can be reversed by PIM447 treatment, proposing the activity of PIM proteins as an essential event to maintain S6 and c-MYC phosphorylation in cells with JAK/STAT pathway mutations. Consequently, the activity of PIM proteins would associate the JAK/STAT pathway with c-MYC and mTOR pathways, which are essential for the onset and progression of many cancer types^45^ and whose deregulation contributes to leukemogenesis^8,9^. In this regard, previous studies have shown that PIM1 can phosphorylate c-MYC at serine 62, increasing its stability and transcriptional capacity^46,47^. Thus, the two signaling pathways most frequently altered in precursor T-cell neoplasms, specifically NOTCH1 and JAK/STAT pathways^10^, could promote c-MYC activation through different mechanisms. In the case of NOTCH1 pathway mutations by inducing c-MYC overexpression, since c-MYC is a transcriptional target of NOTCH1^48,49^, while in the case of JAK/STAT-pathway mutations by leading to c-MYC phosphorylation through the activity of PIM proteins.

Overall, we revealed PIM1 as a potential therapeutic target for the leukemogenic effects mediated by JAK/STAT pathway mutations in precursor T-cell neoplasms. Our results reveal that different JAK/STAT pathway mutations induce *PIM1* overexpression. Moreover, we demonstrate the efficiency of the pan-PIM inhibitor PIM447 against those cells that rely on JAK/STAT pathway mutations recurrently identified in T-ALL/LBL for their growth and viability. The inhibition of PIM proteins by PIM447 would be more feasible, in terms of specificity, than the individualized inhibition of those JAK/STAT pathway members affected by oncogenic mutations, which would presumably require multiple pharmacological inhibitors as well as their combinations. Notably, the inhibition of PIM proteins would also be more efficient than inhibiting mTOR since we observed that treatment with the mTOR inhibitor rapamycin had only a partial effect compared to PIM447 treatment. Moreover, as is the case of STAT proteins, c-MYC is also a transcription factor whose pharmacological inhibition results challenging and no pharmacological inhibitors have been specifically approved against it^50^. Based on the previous reasons, we postulate PIM447 as a potential treatment against mutations affecting different members of the JAK/STAT pathway.

## Methods

### Human samples

Exome and RNA-seq data relative to 274 patients diagnosed with precursor T-cell leukemia/ lymphoma were obtained from tumor samples belonging to the “Therapeutically Applicable Research to Generate Effective Treatments” (TARGET) initiative as well as different biobanks included in the “Spanish Network of Biobanks in Hospitals” (RetBioH; www.redbiobancos.es): Hospital Universitario 12 de Octubre i + 12, Hospital Universitario Ramón y Cajal, Hospital General Universitario Gregorio Marañón, Hospital La Paz, Hospital de Murcia and Fundación Jiménez Díaz. Exome and RNA-seq data from TARGET, accession number phs000218 and sub-study specific accession number phs000464.v19.p8 (TARGET Acute Lymphoblastic Leukemia (ALL) Expansion Phase 2), are managed by the National Cancer Institute (NCI). Information about TARGET can be found at: http://ocg.cancer.gov/programs/target. Data used for this analysis are accessible in the dbGaP database (database of Genotypes and Phenotypes, https://www.ncbi.nlm.nih.gov/projects/gap/cgibin/study.cgi?study_id=phs000464.v19.p8). RNA-seq data relative to normal thymocytes and CD34+ cells were obtained from 3 human postnatal thymuses removed during cardiac surgery in pediatric individuals, as previously described in ref. ^51^.

All samples were collected after written informed consent, according to the declaration of Helsinki, following legal standards for clinical studies in Spain and regulations of the ethics committees from the respective hospitals. The work performed was approved by the Research Ethics Committee of the Universidad Autónoma de Madrid (references CEI31-773 and CEI-70-1260).

### Cell culture

Jurkat (ATCC#TIB-152), MOLT-4 (ATCC# CRL-1582) and HEK293T (ATTC# CRL-11268) cell lines were purchased from ATCC. SUP-T1 (DSMZ#ACC140), HPB-ALL (DSMZ#ACC483), M07e (DSMZ#ACC104) and Ba/F3 (DSMZ#ACC300) cell lines were purchased from the Leibniz Institute-DSMZ. Cell experimentation was always performed within a period not exceeding 6 months after thawing. Cultures were maintained at 37 °C in a humidified atmosphere with 5% CO_2_. Adherent cell lines were grown in Dulbecco’s modified Eagle’s medium (DMEM) (GE Healthcare Life Sciences) supplemented with 10% fetal bovine serum (GE Healthcare Life Sciences), 2 mM L-glutamine (Merck Millipore) and 1 mM sodium pyruvate (Merck Millipore). Suspension cell lines were grown in RPMI 1640 medium (Gibco, Life Technologies) supplemented with 10% fetal bovine serum and 2 mM L-glutamine. ATCC and DSMZ routinely perform cell lines authentication, using short tandem repeat (STR) profiling as a procedure. All cell lines tested negative for mycoplasma contamination.

Due to the lack of appropriate cellular models for the study of JAK/STAT pathway mutations, we made use of M07e and BaF3 hematopoietic cells to generate de novo cellular models that are specifically dependent on different JAK/STAT pathway mutations identified in T-ALL/LBL patients for their growth and viability. M07e and Ba/F3 cells, which require cytokines/growth-factors for normal viability and proliferation, were routinely cultured with 10 ng/ml granulocyte/monocyte colony-stimulating factor (R&DSystems) or 5 ng/ml of interleukin-3 (Cell Signaling), respectively. However, for molecular analysis and functional assays cells were washed 3 times with 1x PBS and seeded in medium without cytokines and growth factors.

### DNA extraction

Total DNA was isolated using the DNeasy 96 Blood and Tissue kit (Qiagen) according to the manufacturer’s instructions. DNA quantification and quality were checked with Nanodrop (Thermo Fisher Scientific Inc.), Qubit (Thermo Fisher Scientific Inc.) and TapeStation (Agilent Technologies).

### Whole exome sequencing (WES)

WES was performed by NIMGenetics SL using the Sure Select All Exome V6 system (Agilent Technologies). Libraries were generated with SureSelectXT Human All Exon V6 technology from Covaris fragmented genomic DNA (150–200 bp). From the amplified libraries, genomic regions of interest were captured using 120 bp RNA probes (SureSelectXT). The generated libraries were normalized and combined in equimolecular concentrations for optimal generation of DNA pools. Pairwise sequencing (2 × 150 bp) of the previously enriched, indexed and multiplexed SureSelectXT libraries was performed on the NovaSeq 6000 platform (Illumina, Inc). Sequencing data were demultiplexed with bcl2fastq2 software (Illumina) and quality was assessed with the FASTQC tool (https://www.bioinformatics.babraham.ac.uk/projects/fastqc/). Alignment was performed using the Burrows-Wheeler Aligner (BWA-MEM) against the GRCh37/hg19 assembly. The results were recalibrated to improve local quality. All these tools are available in the GATK toolkit^52^, and were used following the recommended good practice guide (https://gatk.broadinstitute.org/hc/en-us/sections/360007226651-Best-Practices-Workflows). Variant calling was performed using a combination of the MuTect tool^53^ and VarScan 2^54^. Variant annotation and effect prediction were performed using the ANNOVAR tool^55^, including information from the single nucleotide polymorphism database (dbSNP, build 135), the 1000 Genomes project, the exome variant server (NHLBI GO Exome Sequencing Project, Seattle, WA, USA) and ‘in-house’ scripts.

### Sanger sequencing

DNA fragments amplified by different techniques (PCR products, miniprep, maxiprep, etc) were sent to Macrogen for sequencing, with a final concentration of 100 ng/ml (in the case of vectors) or 20 ng/ml (in the case of PCR amplified products), and together with the corresponding oligonucleotides. The sequences received were analyzed with Chromas Pro (Technelysium Pty Ltd) and aligned against their reference sequence. The oligonucleotides used are summarized in Supplementary Table 4.

### RNA extraction

Total RNA was isolated using the miRNeasy Mini Kit (Qiagen) according to the manufacturer’s instructions. RNA quantification and quality were checked by Nanodrop (Thermo Fisher Scientific Inc.), Qubit (Thermo Fisher Scientific Inc) and TapeStation (Agilent Technologies).

### RNA sequencing (RNA-seq)

RNA-seq was performed by NIMGenetics SL. Using the TruSeq Stranded Total RNA Library Prep (Illumina, Inc.) and included rRNA depletion, fragmentation, cDNA synthesis and adaptor ligation. The generated libraries were normalized and combined in equimolecular concentrations for optimal generation of DNA clusters. Paired-end sequencing (2×100 bp) of the previously enriched, indexed and multiplexed libraries were performed on the high-throughput NovaSeq 6000 platform (Illumina Inc.), with a minimum of 100 M PE reads (50 + 50) per sample, with a read quality of 85% > Q30. For bioinformatics analysis, GRCh38/hg38 (Ensembl version 103) genome was used as a reference. Briefly, quality check and sequence trimming were performed using FASTQC and fastp^56^ respectively. Then, the trimmed RNA-seq reads were aligned against the reference genome and the transcripts were assembled using HISAT2 tool^57^. Corresponding genes were obtained and their expression abundance was determined using StringTie suite (https://ccb.jhu.edu/software/stringtie/). On gene counting matrices, reads were subjected to unsupervised filtering in order to discard those genes with very few or no reads throughout all the samples of the study (https://bioconductor.org/packages/release/bioc/html/genefilter.html). Genes with a total abundance below 15 reads were excluded from further analysis.

### Differential expression analysis

The statistical package DESeq2^58^ was used for differential expression analysis. The differential expression between the two conditions was estimated using the Wald statistic. For multiple testing, the *p*-value was adjusted using the Benjamini-Hochberg procedure^59^.

### Expression analysis by reverse transcription and quantitative PCR (RT-qPCR)

Expression levels for the genes of interest were verified by real-time RT-qPCR from total RNA in two steps. First, using the High-Capacity RNA-to-cDNA™ kit (Applied Biosystems) for reverse transcription (RT), and then using the Fast SYBR® Green Master Mix kit for quantitative PCR (qPCR) in ABI PRISM 7900HT SDS (Applied Biosystems). Expression values obtained for the β2-microglobulin and β-actin genes in the same samples were used for normalization following the 2-ΔΔCT method.

### Gene set enrichment analysis (GSEA)

Gene set enrichment analysis was performed with the Broad Institute’s GSEA program and following the instructions described in the user’s guide^60,61^. All the selected gene sets belong to the molecular signature database (MSigDB) and are summarized in the Supplementary Table 3.

### Pharmacological inhibitors

All inhibitors were purchased from Selleckchem and used in the doses recommended by manufacturers to avoid unspecific cytotoxic effects: the pan-PIM inhibitor PIM447 (#LGH447, 10 µM, 1 µM and 0. 1 µM); the mTOR inhibitor rapamycin (#AY-22989, 100 nM, 50 nM and 10 nM) and the JAK3 inhibitor tofacitinib (#CP-69055 1 µM, 0.5 µM and 0.1 µM). During functional assays involving pharmacological inhibitors, the so-called “untreated cells” were cultured with dimethyl sulfoxide (DMSO) as a negative control.

### Expression vectors

Lentiviral vectors used for expressing, either transiently or stably, the wild-type sequences corresponding to the *JAK1*, *JAK3* and *STAT5* genes were purchased from Vectorbuilder. In such vectors, the expression can be monitored through the levels of Enhanced Green Fluorescent Protein (EGFP). Introduction of the different mutations in the wild-type sequences was performed with the QuickChange Lightning Site-Directed Mutagenesis kit (Agilent Technologies). The previously mentioned vectors were amplified by heat shock transformation of DH5α competent bacteria and subsequent growth in Luria Bertani (LB) medium supplemented with Ampicillin (0.1%) overnight at 37 °C and 225 rpm. Plasmid DNA was then extracted using the Wizard® Plus SV Minipreps DNA purification system (Promega) or GenoPure Plasmid Maxi Kit (Roche Applied Science), depending on the initial culture volume.

Cell transfection was accomplished using Lipofectamine 2000 (Invitrogen) and Opti-MEM medium (Gibco). Cell transduction was performed as previously described^62^. In brief, HEK293T cells, which function as packaging cells, were transfected with the vectors of interest together with psPAX2 and pMD2.6 plasmids. The culture medium of HEK293T cells was replaced after 24 h and, once enriched in the lentiviral particles necessary for transduction, was filtered (Millex), supplemented with polybrene (Sigma-Aldrich) and added to the target cells. To obtain cell populations with comparable expression, transduced cells were sorted for similar EGFP levels using FACS (FACSCVantage SE, BD Biosciences, RRID:SCR_013311).

### Western-Blot (WB)

Protein extraction was performed with RIPA lysis buffer (50 mM Tris-HCl pH 7.4, 150 mM NaCl,1% triton X-100, 0.5% Deoxycholate and 0.1% SDS). Then, proteins extracts were supplemented with 2 mM phenylmethylsulphonyl fluoride, 2.5 μl/ml Protease Inhibitor Cocktail and 10 μl/ml Phosphatase Inhibitor Cocktail 2 (Roche Diagnostics GmbH) as previously described in ref. ^62^. Ten-microgram aliquots of total protein extracts were electrophoresed in 30% acrylamide/bis-acrylamide solution 29:1 (Bio-Rad Laboratories, RRID:SCR_008426) and then electro-transferred to mini-sized nitrocellulose membranes using the Transfer Blot® Turbo™ Transfer System (Bio-Rad Laboratories). Nitrocellulose membranes were incubated with primary and, subsequently, secondary antibodies. Secondary antibodies were conjugated to horseradish peroxidase (GE Healthcare Life Sciences), and the bands were visualized using a cooled charge-coupled device camera (ImageQuant LAS-4000; GE Healthcare Life Sciences). The antibodies used are summarized in Supplementary Table 5. Band densitometry was performed using Image J 1.54i. The results of Western blot band densitometry and statistical analysis are summarized in Supplementary Table 6. For each result, all blots derive from the same experiment and were processed in parallel. Uncropped, unmodified images of every blot included in the figures are shown in Supplementary Fig. 5.

### Functional assays

To analyze growth and viability, cells were counted using trypan blue exclusion and the TC10 Automated Cell Counter (Bio-Rad Laboratories). Functional assays concerning proliferation and cell cycle progression were performed by flow cytometry, using a FACS Canto A cytometer (BD Biosciences), and the obtained results were analyzed with the Flowjo v10 software (LLC). Cell cycle analysis was performed by propidium iodide staining, using the PI/RNase kit (BD Biosciences). Watson pragmatic fitting algorithm was used to determine cell cycle phase statistics using FlowJo v10. Cell proliferation analysis was performed by 5-ethyl-2’-deoxyuridine (EdU) incorporation, using the EdU Staining Proliferation Kit (iFluor 488) (Abcam). Cells were washed, seeded at 500.000 cells/ml and treated with vehicle or the appropriate inhibitor during 96 h for growth and viability experiments or during 24 h for proliferation and cell cycle progression assays. The gating strategy used for Flow cytometry experiments is summarized in Supplementary Fig. 6.

### Statistical analysis

Normality tests were performed using “Shapiro-Wilk test”. The analysis of association between variables was performed using Fisher’s exact test and Chi-square test, as indicated. The differences between independent samples were analyzed using the nonparametric Mann–Whitney test for variables not adjusted to normality. Unpaired two-tailed *t*-test was used for qPCR analysis in Jurkat, MOLT4 and HPB-ALL cell lines and for the rest multiple comparisons were conducted using one- and two-way analysis of variance (ANOVA) with “Dunnett’s multiple comparisons test”. Statistical significance was set at *P* < 0.05. Statistical analyses were performed using the GraphPad Prism, RRID:SCR_002798, version 8. More information about statistical analysis is summarized in Supplementary Table 7.

### Supplementary information

SUPPLEMENTAL MATERIAL

## Data Availability

Data from the Therapeutically Applicable Research to Generate Effective Treatments (TARGET) initiative are managed by the National Cancer Institute (NCI) and accessible through the genotypes and phenotypes database (dbGaP, https://www.ncbi.nlm.nih.gov/projects/gap/cgi-bin/study). RNA-seq data from M07e cells expressing JAK3Q988P are available through the Gene Expression Omnibus database (https://www.ncbi.nlm.nih.gov/geo/) under the accession number GSE266986. Additional data that support the findings of this study are available within the paper and its supplementary information.
