# Supplementary material for: PIM1 is a potential therapeutic target for the leukemogenic effects mediated by JAK/STAT pathway mutations in T-ALL/LBL
Source: NPJ Precis Oncol. 2024 Jul 20;8:152. doi: 10.1038/s41698-024-00638-2 (PMC11271448; doi:10.1038/s41698-024-00638-2)
Supplement: Supplementary file 1 — SUPPLEMENTAL MATERIAL [file 41698_2024_638_MOESM1_ESM.pdf]

SUPPLEMENTAL MATERIAL

Contents:

- **SUPPLEMENTARY TABLES AND LEGENDS**
- **SUPPLEMENTARY FIGURES AND LEGENDS**

SUPPLEMENTARY TABLES AND LEGENDS

Supplementary Table 1. Comparison of multiple clinical features - sex, age at diagnosis, immunophenotype and blasts percentage at diagnosis – between patients with (MUT) and without (WT) JAK/STAT pathway mutations. Male and female frequencies were compared using two-sided Fisher's exact test. Age at diagnosis and immunophenotype were compared using Chi-square test. Mean percentage of blasts at diagnosis was compared using two-tailed Mann Whitney test.

		TOTAL		JAK-STAT_WT		JAK-STAT_MUT		P-VALUE
		N	%	N	%	N	%	
**SEX**	MALE	209	76.8	173	63.6	36	13.2	0.0741
	FEMALE	63	23.2	58	21.3	5	1.8	
	TOTAL	272	100.0	231	84.9	41	15.1	
**AGE AT DIAGNOSIS**	PEDIATRIC (<18)	250	91.6	215	78.8	35	12.8	0.0752
	ADULTS (≥18)	23	8.4	16	5.9	7	2.6	
	TOTAL	273	100.0	231	84.6	42	15.4	
**IMMUNOPHENOTYPE**	PRE-CORTICAL	49	21.1	34	14.7	15	6.5	0.004
	CORTICAL	158	68.1	139	59.9	19	8.2	
	POST-CORTICAL	25	10.8	23	9.9	2	0.9	
	TOTAL	232	100.0	196	84.5	36	15.5	

	TOTAL	JAK-STAT_WT		JAK-STAT_MUT		P-VALUE
	N	N	Mean %	N	Mean %	
**BLASTS AT DIAGNOSIS**	273	231	91.4	42	86.2	0.25

Supplementary Table 2. List of somatic mutations that were found in the patient cohort and that affect JAK/STAT-pathway members.

Mutation	Patient	Gene	Consequence	AA change
1	1	*STAT5B*	missense_variant	N642H
2	1	*JAK1*	missense_variant	E897K
3	2	*JAK3*	missense_variant	M511I
4	3	*IL7R*	inframe_insertion	244-245 -/CPDGR
5	4	*IL7R*	inframe_insertion	244-245 -/CPDGR
6	5	*STAT5B*	missense_variant	N642H
7	6	*STAT5B*	missense_variant	N642H
8	7	*IL7R*	inframe_insertion	243 L/LRGFHITCQ
9	8	*STAT5B*	missense_variant	N418K
10	8	*JAK3*	missense_variant	M511I
11	9	*JAK1*	missense_variant	R724H
12	9	*JAK3*	missense_variant	M511I
13	9	*STAT5B*	missense_variant	T628S
14	10	*JAK3*	missense_variant	P83S
15	11	*JAK3*	missense_variant	M511I
16	12	*JAK1*	missense_variant	R724H
17	12	*JAK3*	missense_variant	R657Q
18	13	*PTPN2*	missense_variant	F71L
19	14	*JAK3*	missense_variant	M511I
20	14	*JAK3*	missense_variant	R887C
21	15	*STAT5B*	missense_variant	S434L
22	16	*IL7R*	inframe_insertion	243 L/LQRCT
23	17	*IL7R*	inframe_insertion	242 L/FCTPVP
24	18	*JAK1*	missense_variant	R724H
25	18	*JAK3*	missense_variant	V674A
26	19	*PTPN2*	missense_variant	T50A
27	20	*JAK3*	missense_variant	V678M
28	20	*JAK3*	missense_variant	M511I
29	21	*PTPRC*	stop_gained	592 R/RA*
30	22	*JAK3*	missense_variant	V674A
31	22	*STAT5B*	missense_variant	N642H
32	23	*STAT5B*	missense_variant	D658Y
33	24	*PTPRC*	missense_variant	G704R
34	25	*IL7R*	inframe_insertion	255 L/LFLE
35	26	*JAK3*	missense_variant	L857P
36	26	*STAT5B*	missense_variant	N642H
37	27	*JAK3*	missense_variant	V674A
38	28	*STAT5B*	missense_variant	V712E
39	29	*JAK1*	missense_variant	R724H
40	29	*JAK1*	missense_variant	Y652H
41	29	*JAK3*	missense_variant	M511I
42	30	*JAK1*	missense_variant	F734L
43	30	*JAK3*	missense_variant	L857P

44	31	*JAK1*	missense_variant	F838L
45	31	*JAK3*	missense_variant	R657Q
46	31	*PTPRC*	frameshift_variant	299 H/QX
47	32	*STAT5B*	missense_variant	N642H
48	33	*IL7R*	inframe_insertion	242 L/LCPS
49	34	*IL7R*	inframe_insertion	244 T/RRCSS
50	35	*IL7R*	inframe_insertion	242 -/RR
51	35	*JAK3*	missense_variant	M511I
52	36	*JAK1*	missense_variant	F805V
53	36	*JAK1*	missense_variant	R724H
54	36	*JAK3*	missense_variant	L857P
55	36	*STAT5B*	missense_variant	I704L
56	37	*STAT5A*	missense_variant	D658N
57	37	*JAK3*	missense_variant	G292A
58	38	*STAT5B*	missense_variant	N642H
59	38	*STAT5B*	missense_variant	G698E
60	39	*JAK1*	missense_variant	R724H
61	39	*STAT5B*	missense_variant	N642H
62	40	*JAK3*	missense_variant	M511I
63	40	*JAK3*	missense_variant	Q988P
64	41	*PTPRC*	frameshift_variant	652L/X
65	41	*PTPRC*	frameshift_variant	664F/LX
66	41	*PTPN2*	missense_variant	T42A
67	42	*JAK3*	missense_variant	M511I

Supplementary Table 3. List of gene sets for GSEA analysis employed during the study, indicating: the systematic name, collection, and size.

	Signature name	Collection	ID	Size
Pathway	c-MYC	Hallmark	M5926	200
	JAK/STAT	Hallmark	M5947	200
	MAPK/ERK	Hallmark	M5953	200
	mTOR	Hallmark	M5924	200
	NOTCH1	Hallmark	M5903	32
	PI3K/AKT	Hallmark	M5923	105
Cellular process	Adipogenesis	Hallmark	M5905	200
	Autophagy	KEGG	M27935	151
	Cell to cell communication	Reactome	M522	133
	Chromosome maintenance	Reactome	M868	140
	DNA repair	Reactome	M15434	333
	Extracellular matrix organization	Reactome	M610	300
	G2/M checkpoint damage	Reactome	M1080	106
	Glycolisis	Hallmark	M5937	200
	Hypoxia	Hallmark	M5891	200
	Mitotic prophase	Reactome	M27660	143
	Oxidative phosphorylation	Hallmark	M5936	200
	P53 signaling	Hallmark	M5939	200
	Protein export	KEGG	M6981	24
	Protein folding	Reactome	M776	98
	Protein secretion	Hallmark	M5910	96
	Regulation of Actin cytoskeleton	KEGG	M18306	213
	rRNA processing	Reactome	M27685	205
	Senescence	Reactome	M27188	198
	Steroid Biosynthesis	KEGG	M5872	17
	Telomere maintenance	Reactome	M4052	113

Supplementary Table 4. List of oligonucleotides employed during the study, indicating: the application, gene name, identification, and sequence.

Application	Gene	Oligonucleotide	Sequence (5´ > 3´)
qPCR	*ACTB*	ACTB_Fw	AGTGTGACGTGGACATCCGCAAAG
		ACTB_Rv	ATCCACATCTGCTGGAAGGTGGAC
	B2M	B2M_Fw	CCAGCAGAGAATGGAAAGTC
		B2M_Rv	GATGCTGCTTACATGTCTCG
	PIM1	PIM1_Fw	CTCAATCGCGGCGAGCTCAA
		PIM1_Rv	CCATGGTAGCGATGGTAGCGGA
		mPIM1_Fw	TCTTAATCGACCTGAGCCGC
		mPIM1_Rv	GTGGTAGCGATGGTAGCGAA
Mutagenesis	*JAK1*	Jak1 1957C>T_Fw	GACACAGACGCCATAGAAGTACACGATGTGTTTGT
		Jak1 1957C>T_Rv	ACAAACACATCGTGTACTTCTATGGCGTCTGTGTC
	JAK3	JAK3 2963A>C_Fw	GAAAATGGGGCTCGGGCCTGGCTCGCG
		JAK3 2963A>C_Rv	CGCGAGCCAGGCCCGAGCCCCATTTTC
	STAT5	STAT5 1924A>C_Fw	GGTGGTAAAAGGCATCAGATGCCAAAACATTCTTTCCTGAG
		STAT5 1924A>C_Rv	CTCAGGAAAGAATGTTTTGGCATCTGATGCCTTTTACCACC

Supplementary Table 5. List of antibodies employed during the study, indicating: the commercial name, dilution, specie, company, and catalog number.

Antibody	Dilution	Species	Company, catalog No
NOTCH1 (D6F11)	1/1000	Rabbit	Cell Signaling, 4380
cleaved NOTCH1 (Val1744) (D3B8)	1/1000	Rabbit	Cell Signaling, 4147
AKT	1/1000	Rabbit	Cell Signaling, 9272
P-AKT (Thr 308) (D25E6)	1/1000	Rabbit	Cell Signaling, 13038
P-AKT1 (Ser 473) (D7F10)	1/500	Rabbit	Cell Signaling, 9018
p44/42 MAPK (Erk1/2)	1/1000	Rabbit	Cell Signaling, 9102
P-p44/42 MAPK (Erk1/2) (Thr202/Tyr204)	1/1000	Rabbit	Cell Signaling, 9101
JAK1 (6G4)	1/1000	Rabbit	Cell Signaling, 3344
JAK3 (D7B12)	1/1000	Rabbit	Cell Signaling, 8863
STAT1	1/1000	Rabbit	Cell Signaling, 9172
P-STAT1 (Tyr701) (D4A7)	1/1000	Rabbit	Cell Signaling, 7649
STAT5 (D2O6Y)	1/1000	Rabbit	Cell Signaling, 94205
P-STAT5 (Tyr694)	1/1000	Mouse	BD Biosciences, 611965
PIM1 (C93F2)	1/1000	Rabbit	Cell Signaling, 3247
PIM2 (D1D2)	1/1000	Rabbit	Cell Signaling, 4730
PIM3 (D17C9)	1/1000	Rabbit	Cell Signaling, 4165
c-MYC (D84C12)	1/1000	Rabbit	Cell Signaling, 5605
P-c-MYC (Ser62) (E1J4K)	1/1000	Rabbit	Cell Signaling, 13748
S6 RP (5G10)	1/1000	Rabbit	Cell Signaling, 2217
P-S6 RP (Ser235/236) (D57.2.2E)	1/1000	Rabbit	Cell Signaling, 4858
4EBP1 (53H11)	1/1000	Rabbit	Cell Signaling, 9644
P-4EBP1 (Thr37/46) (236B4)	1/1000	Rabbit	Cell Signaling, 2855
Cyclin D2 (D52F9)	1/1000	Rabbit	Cell Signaling, 3741
β-Actin (AC-15)	1/20000	Mouse	Sigma Aldrich, A5441
HRP-anti-mouse igG antibody	1/1000	-	Cell Signaling, 7076
HRP-anti-rabbit igG antibody	1/1000	-	Cell Signaling, 7074

Supplementary Table 6. Summary of the densitometry analysis performed in each of the Western blot figures. Details of the densitometry analysis are shown: the test performed, the mean and standard deviation (s.d) and the control lane against which comparisons were made. For each of the three replicates, protein bands for PIM1, cyclinD2, c-MYC, p-c-MYC, p-S6 and p-4EBP1 were referred to the corresponding actin band and normalized against the control lane.

One-way ANOVA: Dunnett's multiple comparisons test				
**Figure 2D**				
**M07e J3Q988P**				
**PIM1**	**mean**	**s.d**	**Adjusted p-value**	**Comparisons**
**0**	1.0000	0.0000		Statistical comparisons are made against non-transduced cells (0)
**WT**	0.8626	0.0250	0.2184	
**Q988P**	4.8607	0.1257	<0.0001	
**M07e J1V658F**				
**PIM1**	**mean**	**s.d**	**Adjusted p-value**	
**0**	1.0000	0.0000		
**WT**	1.0501	0.3390	0.9510	
**V658F**	17.4838	0.4598	<0.0001	
**M07e S5N642H**				
**PIM1**	**mean**	**s.d**	**Adjusted p-value**	
**0**	1.0000	0.0000		
**WT**	0.9407	0.0603	0.4376	
**N642H**	6.2527	0.1500	<0.0001	
**Figure 2F**				
**PIM1**	**mean**	**s.d**	**Adjusted p-value**	**Comparisons**
**M07e-J3-Q988P**	1.0000	0.0000		Statistical comparisons are made against M07e-J3-Q988P
**Jurkat**	0.0868	0.0008	<0.0001	
**HPB-ALL**	0.0903	0.0024	<0.0001	
**SUP-T1**	0.0472	0.0005	<0.0001	
**Figure 2D**				
**M07e J3Q988P**				
**PIM1**	**mean**	**s.d**	**Adjusted p-value**	**Comparisons**
**Tofacitinib 0**	1.0000	0.0000		Statistical comparisons are made against untreated cells (Tofactinib 0)
**Tofacitinib 0.1µM**	0.5320	0.0068	<0.0001	
**Tofacitinib 0.5µM**	0.3538	0.0003	<0.0001	
**Tofacitinib 1µM**	0.2964	0.0058	<0.0001	
**Figure 3G**				
**M07e J3Q988P**				
**Cyclin D2**	**mean**	**s.d**	**Adjusted p-value**	**Comparisons**
**Untreated**	1.0000	0.0000		Statistical comparisons are made against untreated cells
**PIM447**	0.5540	0.0022	<0.0001	
**M07e J1V658F**				
**Cyclin D2**	**mean**	**s.d**	**Adjusted p-value**	
**Untreated**	1.0000	0.0000		
**PIM447**	0.5439	0.0168	<0.0001	

M07e S5N642H				
Cyclin D2	mean	s.d	Adjusted p-value	
Untreated	1.0000	0.0000		
PIM447	0.4331	0.0121	<0.0001	
Ba/F3 J3Q988P				
Cyclin D2	mean	s.d	Adjusted p-value	
Untreated	1.0000	0.0000		
PIM447	0.4437	0.0051	<0.0001	
Ba/F3 J1V658F				
Cyclin D2	mean	s.d	Adjusted p-value	
Untreated	1.0000	0.0000		
PIM447	0.6682	0.0164	<0.0001	
Ba/F3 S5N642H				
Cyclin D2	mean	s.d	Adjusted p-value	
Untreated	1.0000	0.0000		
PIM447	0.4634	0.0184	<0.0001	
Figure 4D				
M07e J3Q988P				
c-MYC	mean	s.d	Adjusted p-value	Comparisons
PIM447 0	1.0000	0.0000		Statistical comparisons are made against untreated cells (PIM447 0)
PIM447 0.1µM	0.9212	0.0293	0.0007	
PIM447 1µM	0.7166	0.0042	<0.0001	
PIM447 10µM	0.4995	0.0108	<0.0001	
p-c-MYC	mean	s.d	Adjusted p-value	
PIM447 0	1.0000	0.0000		
PIM447 0.1µM	0.9876	0.0054	0.1958	
PIM447 1µM	0.6967	0.0041	<0.0001	
PIM447 10µM	0.5457	0.0118	<0.0001	
p-S6	mean	s.d	Adjusted p-value	
PIM447 0	1.0000	0.0000		
PIM447 0.1µM	0.7849	0.0043	<0.0001	
PIM447 1µM	0.4693	0.0028	<0.0001	
PIM447 10µM	0.2555	0.0055	<0.0001	
M07e J1V658F				
c-MYC	mean	s.d	Adjusted p-value	
PIM447 0	1.0000	0.0000		
PIM447 0.1µM	0.8490	0.0031	<0.0001	
PIM447 1µM	0.6920	0.0272	<0.0001	
PIM447 10µM	0.4927	0.0246	<0.0001	
p-c-MYC	mean	s.d	Adjusted p-value	
PIM447 0	1.0000	0.0000		
PIM447 0.1µM	0.8015	0.0029	<0.0001	
PIM447 1µM	0.6732	0.0265	<0.0001	

**PIM447 10µM**	0.5114	0.0255	<0.0001	
**p-S6**	**mean**	**s.d**	**Adjusted p-value**	
**PIM447 0**	1.0000	0.0000		
**PIM447 0.1µM**	0.4959	0.0018	<0.0001	
**PIM447 1µM**	0.1592	0.0063	<0.0001	
**PIM447 10µM**	0.0609	0.0030	<0.0001	
**M07e S5N642H**				
**c-MYC**	**mean**	**s.d**	**Adjusted p-value**	
**PIM447 0**	1.0000	0.0000		
**PIM447 0.1µM**	0.8504	0.0159	<0.0001	
**PIM447 1µM**	0.7383	0.0013	<0.0001	
**PIM447 10µM**	0.4164	0.0103	<0.0001	
**p-c-MYC**	**mean**	**s.d**	**Adjusted p-value**	
**PIM447 0**	1.0000	0.0000		
**PIM447 0.1µM**	0.7596	0.0142	<0.0001	
**PIM447 1µM**	0.6420	0.0011	<0.0001	
**PIM447 10µM**	0.3909	0.0097	<0.0001	
**p-S6**	**mean**	**s.d**	**Adjusted p-value**	
**PIM447 0**	1.0000	0.0000		
**PIM447 0.1µM**	0.6341	0.0119	<0.0001	
**PIM447 1µM**	0.1851	0.0003	<0.0001	
**PIM447 10µM**	0.0671	0.0017	<0.0001	
**Jurkat**				
**c-MYC**	**mean**	**s.d**	**Adjusted p-value**	
**PIM447 0**	1.0000	0.0000		
**PIM447 0.1µM**	0.9281	0.0260	0.9045	
**PIM447 1µM**	0.9425	0.1680	0.9462	
**PIM447 10µM**	0.9600	0.2756	0.9802	
**p-c-MYC**	**mean**	**s.d**	**Adjusted p-value**	
**PIM447 0**	1.0000	0.0000		
**PIM447 0.1µM**	0.9893	0.0277	0.9519	
**PIM447 1µM**	1.0077	0.0256	0.9806	
**PIM447 10µM**	0.9372	0.0504	0.0959	
**p-S6**	**mean**	**s.d**	**Adjusted p-value**	
**PIM447 0**	1.0000	0.0000		
**PIM447 0.1µM**	0.9685	0.0272	0.5362	
**PIM447 1µM**	0.9275	0.0235	0.0645	
**PIM447 10µM**	1.0194	0.0548	0.8121	
**Figure 4F**				
**M07e**				
**c-MYC**	**mean**	**s.d**	**Adjusted p-value**	**Comparisons**
**JAK3 -**	1.0000	0.0000		Statistical comparisons are
**JAK3 WT**	0.9177	0.0431	0.4381	

JAK3 Q988P	2.6694	0.1409	<0.0001	made against non-transduced cells (JAK3 -)
**p-c-MYC**	**mean**	**s.d**	**Adjusted p-value**	
**JAK3 -**	1.0000	0.0000		
**JAK3 WT**	0.8783	0.0318	0.1093	
**JAK3 Q988P**	2.0728	0.1094	<0.0001	
**p-S6**	**mean**	**s.d**	**Adjusted p-value**	
**JAK3 -**	1.0000	0.0000		
**JAK3 WT**	1.0822	0.0348	0.9277	
**JAK3 Q988P**	10.1977	0.5384	<0.0001	
**Ba/F3**				
**c-MYC**	**mean**	**s.d**	**Adjusted p-value**	
**JAK3 -**	1.0000	0.0000		
**JAK3 WT**	0.9695	0.0340	0.9618	
**JAK3 Q988P**	3.3999	0.2772	<0.0001	
**p-c-MYC**	**mean**	**s.d**	**Adjusted p-value**	
**JAK3 -**	1.0000	0.0000		
**JAK3 WT**	1.0826	0.0379	0.7806	
**JAK3 Q988P**	3.5401	0.2886	<0.0001	
**p-S6**	**mean**	**s.d**	**Adjusted p-value**	
**JAK3 -**	1.0000	0.0000		
**JAK3 WT**	1.0497	0.0368	0.9053	
**JAK3 Q988P**	3.4347	0.2800	<0.0001	
**Figure 5C**				
**M07e J3Q988P**				
**p-S6**	**mean**	**s.d**	**Adjusted p-value**	**Comparisons**
**Rapamycin 0**	1.0000	0.0000		Statistical comparisons are made against untreated cells (Rapamycin 0)
**Rapamycin 10nM**	0.1973	0.0000	<0.0001	
**Rapamycin 50nM**	0.1227	0.0025	<0.0001	
**Rapamycin 100nM**	0.1003	0.0048	<0.0001	
**p-4EBP1**	**mean**	**s.d**	**Adjusted p-value**	
**Rapamycin 0**	1.0000	0.0000		
**Rapamycin 10nM**	0.8807	0.0001	<0.0001	
**Rapamycin 50nM**	0.6633	0.0137	<0.0001	
**Rapamycin 100nM**	0.5221	0.0252	<0.0001	
**M07e J1V658F**				
**p-S6**	**mean**	**s.d**	**Adjusted p-value**	
**Rapamycin 0**	1.0000	0.0000		
**Rapamycin 10nM**	0.0741	0.0014	<0.0001	
**Rapamycin 50nM**	0.0404	0.0008	<0.0001	
**Rapamycin 100nM**	0.0478	0.0005	<0.0001	
**p-4EBP1**	**mean**	**s.d**	**Adjusted p-value**	
**Rapamycin 0**	1.0000	0.0000		
**Rapamycin 10nM**	0.7717	0.0143	<0.0001	

**Rapamycin 50nM**	0.7774	0.0148	<0.0001	
**Rapamycin 100nM**	0.6778	0.0075	<0.0001	
**M07e S5N642H**				
**p-S6**	**mean**	**s.d**	**Adjusted p-value**	
**Rapamycin 0**	1.0000	0.0000		
**Rapamycin 10nM**	0.1134	0.0021	<0.0001	
**Rapamycin 50nM**	0.0256	0.0009	<0.0001	
**Rapamycin 100nM**	0.0572	0.0020	<0.0001	
**p-4EBP1**	**mean**	**s.d**	**Adjusted p-value**	
**Rapamycin 0**	1.0000	0.0000		
**Rapamycin 10nM**	0.7219	0.0136	<0.0001	
**Rapamycin 50nM**	0.7213	0.0254	<0.0001	
**Rapamycin 100nM**	0.6568	0.0234	<0.0001	
**Figure S2B**				
**Ba/F3 J3Q988P**				
**PIM1**	**mean**	**s.d**	**Adjusted p-value**	**Comparisons**
**0**	1.0000	0.0000		Statistical comparisons are made against non-transduced cells (0)
**WT**	1.0803	0.5018	0.9111	
**Q988P**	34.6660	0.3713	<0.0001	
**Ba/F3 J1V658F**				
**PIM1**	**mean**	**s.d**	**Adjusted p-value**	
**0**	1.0000	0.0000		
**WT**	0.9016	0.2968	0.9723	
**V658F**	24.0128	1.5130	<0.0001	
**Ba/F3 S5N642H**				
**PIM1**	**mean**	**s.d**	**Adjusted p-value**	
**0**	1.0000	0.0000		
**WT**	1.1170	0.0757	0.9144	
**N642H**	9.2945	0.4432	<0.0001	
**Figure S3A**				
**Ba/F3 J3Q988P**				
**c-MYC**	**mean**	**s.d**	**Adjusted p-value**	**Comparisons**
**PIM447 0**	1.0000	0.0000		Statistical comparisons are made against untreated cells (PIM447 0)
**PIM447 0.1μM**	0.9982	0.0034	0.9421	
**PIM447 1μM**	0.7074	0.0068	<0.0001	
**PIM447 10μM**	0.3795	0.0063	<0.0001	
**p-c-MYC**	**mean**	**s.d**	**Adjusted p-value**	
**PIM447 0**	1.0000	0.0000		
**PIM447 0.1μM**	0.9943	0.0034	0.3201	
**PIM447 1μM**	0.7561	0.0073	<0.0001	
**PIM447 10μM**	0.2083	0.0034	<0.0001	
**p-S6**	**mean**	**s.d**	**Adjusted p-value**	
**PIM447 0**	1.0000	0.0000		

**PIM447 0.1µM**	0.7380	0.0025	<0.0001	
**PIM447 1µM**	0.3086	0.0030	<0.0001	
**PIM447 10µM**	0.1722	0.0028	<0.0001	
**Ba/F3 J1V658F**				
**c-MYC**	**mean**	**s.d**	**Adjusted p-value**	
**PIM447 0**	1.0000	0.0000		
**PIM447 0.1µM**	0.9813	0.0134	0.1357	
**PIM447 1µM**	0.7066	0.0079	<0.0001	
**PIM447 10µM**	0.4077	0.0138	<0.0001	
**p-c-MYC**	**mean**	**s.d**	**Adjusted p-value**	
**PIM447 0**	1.0000	0.0000		
**PIM447 0.1µM**	1.0165	0.0138	0.2021	
**PIM447 1µM**	0.8128	0.0091	<0.0001	
**PIM447 10µM**	0.3789	0.0128	<0.0001	
**p-S6**	**mean**	**s.d**	**Adjusted p-value**	
**PIM447 0**	1.0000	0.0000		
**PIM447 0.1µM**	0.7707	0.0105	<0.0001	
**PIM447 1µM**	0.3895	0.0044	<0.0001	
**PIM447 10µM**	0.2294	0.0078	<0.0001	
**Ba/F3 S5N642H**				
**c-MYC**	**mean**	**s.d**	**Adjusted p-value**	
**PIM447 0**	1.0000	0.0000		
**PIM447 0.1µM**	0.8419	0.0022	<0.0001	
**PIM447 1µM**	0.7023	0.0349	<0.0001	
**PIM447 10µM**	0.4042	0.0085	<0.0001	
**p-c-MYC**	**mean**	**s.d**	**Adjusted p-value**	
**PIM447 0**	1.0000	0.0000		
**PIM447 0.1µM**	0.7212	0.0019	<0.0001	
**PIM447 1µM**	0.5832	0.0290	<0.0001	
**PIM447 10µM**	0.3615	0.0076	<0.0001	
**p-S6**	**mean**	**s.d**	**Adjusted p-value**	
**PIM447 0**	1.0000	0.0000		
**PIM447 0.1µM**	0.6574	0.0017	<0.0001	
**PIM447 1µM**	0.1532	0.0076	<0.0001	
**PIM447 10µM**	0.0264	0.0006	<0.0001	
**Figure S4B**				
**Ba/F3 J3Q988P**				
**p-S6**	**mean**	**s.d**	**Adjusted p-value**	**Comparisons**
**Rapamycin 0**	1.0000	0.0000		Statistical comparisons are made against untreated cells (Rapamycin 0)
**Rapamycin 10nM**	0.3467	0.0015	<0.0001	
**Rapamycin 50nM**	0.3072	0.0006	<0.0001	
**Rapamycin 100nM**	0.2172	0.0059	<0.0001	
**p-4EBP1**	**mean**	**s.d**	**Adjusted p-value**	

**Rapamycin 0**	1.0000	0.0000	
**Rapamycin 10nM**	0.7569	0.0032	<0.0001
**Rapamycin 50nM**	0.7391	0.0298	<0.0001
**Rapamycin 100nM**	0.5692	0.0154	<0.0001
**Ba/F3 J1V658F**			
**p-S6**	**mean**	**s.d**	**Adjusted p-value**
**Rapamycin 0**	1.0000	0.0000	
**Rapamycin 10nM**	0.2047	0.0008	<0.0001
**Rapamycin 50nM**	0.2018	0.0022	<0.0001
**Rapamycin 100nM**	0.1401	0.0005	<0.0001
**p-4EBP1**	**mean**	**s.d**	**Adjusted p-value**
**Rapamycin 0**	1.0000	0.0000	
**Rapamycin 10nM**	0.6246	0.0026	<0.0001
**Rapamycin 50nM**	0.5899	0.0064	<0.0001
**Rapamycin 100nM**	0.4675	0.0018	<0.0001
**Ba/F3 S5N642H**			
**p-S6**	**mean**	**s.d**	**Adjusted p-value**
**Rapamycin 0**	1.0000	0.0000	
**Rapamycin 10nM**	0.1936	0.0072	<0.0001
**Rapamycin 50nM**	0.1497	0.0038	<0.0001
**Rapamycin 100nM**	0.1116	0.0005	<0.0001
**p-4EBP1**	**mean**	**s.d**	**Adjusted p-value**
**Rapamycin 0**	1.0000	0.0000	
**Rapamycin 10nM**	0.6037	0.0225	<0.0001
**Rapamycin 50nM**	0.6015	0.0154	<0.0001
**Rapamycin 100nM**	0.3970	0.0017	<0.0001

Supplementary Table 7. Summary of the statistical analysis performed in each figure. Details of the statistical analyses are shown, including: the tests performed, the mean and standard deviation (s.d.), the confidence intervals (CI) of the difference at 95%, and the corresponding P values or adjusted P values.

**Figure 2C**	**One-way ANOVA; Dunnett's multiple comparisons test**						
**M07e**	**Mean 1**	**Mean 2**	**s.d 1**	**s.d 2**	**Mean Difference**	**95% CI of difference**	**Adjusted P Value**
**Q988P**							
0 vs WT	1.0070	1.1970	0.0379	0.0651	-0.1900	-0.5879 to 0.2079	0.3520
0 vs Q988P	1.0070	8.2630	0.0379	0.2850	-7.2570	-7.655 to -6.859	<0.0001
**V658F**							
0 vs WT	0.9967	1.0770	0.0208	0.0321	-0.0800	-0.2732 to 0.1132	0.4376
0 vs V658F	0.9967	7.3070	0.0208	0.1380	-6.3100	-6,503 to -6,117	<0.0001
**N642H**							
0 vs WT	1.0000	1.0370	0.0608	0.0603	-0.0367	-1.742 to 1.669	0.9972
0 vs N642H	1.0000	4.9100	0.0608	1.2605	-3.9100	-5.615 to -2.205	0.0011
**Figure 2E**	**One-way ANOVA; Dunnett's multiple comparisons test**						
	**Mean 1**	**Mean 2**	**s.d 1**	**s.d 2**	**Mean Difference**	**95% CI of difference**	**Adjusted P Value**
M07e-J3-Q988P vs. Jurkat	1.0000	0.0233	0.0200	0.0058	0.9767	0.9504 to 1.003	<0.0001
M07e-J3-Q988P vs. HPB-ALL	1.0000	0.0567	0.0200	0.0058	0.9433	0.9170 to 0.9696	<0.0001
M07e-J3-Q988P vs. SUPT-1	1.0000	0.0567	0.0200	0.0058	0.9433	0.9170 to 0.9696	<0.0001
**Figure 2G**	**One-way ANOVA; Dunnett's multiple comparisons test**						
	**Mean 1**	**Mean 2**	**s.d 1**	**s.d 2**	**Mean Difference**	**95% CI of difference**	**Adjusted P Value**
PIM1							
M07e vs M07e-J3-Q988P	1.0000	3.9700	0.1130	1.0959	-2.9700	-4.072 to -1.867	<0.0001
PIM2							
M07e vs M07e-J3-Q988P	1.0000	1.4250	0.1328	0.3505	-0.4250	-1.527 to 0.6771	0.6663
PIM3							
M07e vs M07e-J3-Q988P	1.0000	0.9271	0.0976	0.2463	0.0729	-1.029 to 1.175	0.9971

Figure 3A	Two-way ANOVA; Dunnett's multiple comparisons test						
**M07e cell growth**	**Mean 1**	**Mean 2**	**s.d 1**	**s.d 2**	**Mean Difference**	**95% CI of difference**	**Adjusted P Value**
**Q988P**							
0 vs. 0.1	1.0000	0.6477	0.0537	0.1218	0.3523	0.2119 to 0.4927	<0.0001
0 vs. 1	1.0000	0.2133	0.0537	0.0521	0.7867	0.6463 to 0.9271	<0.0001
0 vs. 10	1.0000	0.1235	0.0537	0.0339	0.8765	0.7361 to 1.017	<0.0001
**V658F**							
0 vs. 0.1	1.0000	0.7679	0.2134	0.0983	0.2321	0.09170 to 0.3725	0.0010
0 vs. 1	1.0000	0.2906	0.2134	0.0497	0.7094	0.5690 to 0.8498	<0.0001
0 vs. 10	1.0000	0.1222	0.2134	0.0132	0.8778	0.7374 to 1.018	<0.0001
**N642H**							
0 vs. 0.1	1.0000	0.7085	0.0349	0.0667	0.2915	0.1510 to 0.4319	<0.0001
0 vs. 1	1.0000	0.2834	0.0349	0.0526	0.7166	0.5762 to 0.8570	<0.0001
0 vs. 10	1.0000	0.1116	0.0349	0.0138	0.8884	0.7480 to 1.029	<0.0001
**Jurkat**							
0 vs. 0.1	1.0000	0.9533	0.0298	0.0237	0.0467	-0.09373 to 0.1871	0.7427
0 vs. 1	1.0000	0.9894	0.0298	0.0271	0.0107	-0.1298 to 0.1511	0.9950
0 vs. 10	1.0000	0.9443	0.0298	0.0558	0.0557	-0.08472 to 0.1961	0.6352
**Ba/F3 cell growth**	**Mean 1**	**Mean 2**	**s.d 1**	**s.d 2**	**Mean Difference**	**95% CI of difference**	**Adjusted P Value**
**Q988P**							
0 vs. 0.1	1.0000	0.3431	0.0582	0.0620	0.6569	0.5728 to 0.7410	<0.0001
0 vs. 1	1.0000	0.1356	0.0582	0.0340	0.8644	0.7803 to 0.9485	<0.0001
0 vs. 10	1.0000	0.0909	0.0582	0.0203	0.9091	0.8250 to 0.9932	<0.0001
**V658F**							
0 vs. 0.1	1.0000	0.3484	0.0420	0.0743	0.6516	0.5675 to 0.7357	<0.0001
0 vs. 1	1.0000	0.1565	0.0420	0.0137	0.8435	0.7594 to 0.9276	<0.0001
0 vs. 10	1.0000	0.0887	0.0420	0.0077	0.9113	0.8273 to 0.9954	<0.0001

**N642H**							
0 vs. 0.1	1.0000	0.3613	0.0214	0.0568	0.6387	0.5546 to 0.7228	<0.0001
0 vs. 1	1.0000	0.1484	0.0214	0.0131	0.8516	0.7675 to 0.9357	<0.0001
0 vs. 10	1.0000	0.0922	0.0214	0.0203	0.9078	0.8237 to 0.9919	<0.0001
**Jurkat**							
0 vs. 0.1	1.0000	1.0270	0.0356	0.0052	-0.02743	-0.1115 to 0.05666	0.7529
0 vs. 1	1.0000	0.9886	0.0356	0.0510	0.01139	-0.07271 to 0.09548	0.9737
0 vs. 10	1.0000	0.9800	0.0356	0.0328	0.02001	-0.06408 to 0.1041	0.8810
**Figure 3B**	**Two-way ANOVA; Dunnett's multiple comparisons test**						
**M07e viability**	**Mean 1**	**Mean 2**	**s.d 1**	**s.d 2**	**Mean Difference**	**95% CI of difference**	**Adjusted P Value**
**Q988P**							
0 vs. 0.1	1.000	0.9573	0.0269	0.0163	0.0427	-0.04311 to 0.1285	0.4783
0 vs. 1	1.000	0.726	0.0269	0.0847	0.274	0.1882 to 0.3598	<0.0001
0 vs. 10	1.000	0.4128	0.0269	0.0163	0.5872	0.5014 to 0.6730	<0.0001
**V658F**							
0 vs. 0.1	1.000	0.9713	0.0372	0.0248	0.02867	-0.05714 to 0.1145	0.7482
0 vs. 1	1.000	0.7993	0.0372	0.0657	0.2007	0.1149 to 0.2865	<0.0001
0 vs. 10	1.000	0.4373	0.0372	0.0271	0.5627	0.4769 to 0.6485	<0.0001
**N642H**							
0 vs. 0.1	1.000	0.9854	0.0334	0.0190	0.0146	-0.07121 to 0.1004	0.9528
0 vs. 1	1.000	0.8139	0.0334	0.0669	0.1861	0.1003 to 0.2719	<0.0001
0 vs. 10	1.000	0.4672	0.0334	0.0228	0.5328	0.4470 to 0.6187	<0.0001
**Jurkat**							
0 vs. 0.1	1.000	0.9521	0.0214	0.0729	0.04795	-0.03787 to 0.1338	0.3874
0 vs. 1	1.000	0.9384	0.0214	0.0259	0.06164	-0.02417 to 0.1475	0.2036
0 vs. 10	1.000	0.9726	0.0214	0.0259	0.0274	-0.05842 to 0.1132	0.7719
**Ba/F3 cell viability**	**Mean 1**	**Mean 2**	**s.d 1**	**s.d 2**	**Mean Difference**	**95% CI of difference**	**Adjusted P Value**

**Q988P**							
0 vs. 0.1	1.0000	0.9759	0.0273	0.0157	0.02405	-0.06853 to 0.1166	0.8526
0 vs. 1	1.0000	0.8247	0.0273	0.0574	0.1753	0.08267 to 0.2678	0.0002
0 vs. 10	1.0000	0.567	0.0273	0.0627	0.433	0.3404 to 0.5256	<0.0001
**V658F**							
0 vs. 0.1	1.0000	0.9761	0.0156	0.0156	0.02389	-0.06870 to 0.1165	0.855
0 vs. 1	1.0000	0.7918	0.0156	0.0726	0.2082	0.1156 to 0.3008	<0.0001
0 vs. 10	1.0000	0.5324	0.0156	0.0369	0.4676	0.3750 to 0.5602	<0.0001
**N642H**							
0 vs. 0.1	1.0000	0.9552	0.0333	0.0333	0.04483	-0.04776 to 0.1374	0.49
0 vs. 1	1.0000	0.8103	0.0333	0.0363	0.1897	0.09707 to 0.2822	<0.0001
0 vs. 10	1.0000	0.4966	0.0333	0.0724	0.5034	0.4109 to 0.5960	<0.0001
**Jurkat**							
0 vs. 0.1	1.0000	0.9381	0.0179	0.0273	0.06186	-0.03073 to 0.1544	0.2466
0 vs. 1	1.0000	0.9622	0.0179	0.0390	0.0378	-0.05479 to 0.1304	0.6155
0 vs. 10	1.0000	0.9828	0.0179	0.0298	0.01718	-0.07540 to 0.1098	0.9373
**Figure 3C**	**One-way ANOVA; Dunnett's multiple comparisons test**						
**M07e**	**Mean 1**	**Mean 2**	**s.d 1**	**s.d 2**	**Mean Difference**	**95% CI of difference**	**Adjusted P Value**
Jurkat vs. V658F	0.9944	0.5851	0.0124	0.0106	0.4093	0.3658 to 0.4529	<0.0001
Jurkat vs. Q988P	0.9944	0.6027	0.0124	0.0201	0.3917	0.3482 to 0.4353	<0.0001
Jurkat vs. N642H	0.9944	0.5075	0.0124	0.0265	0.4869	0.4434 to 0.5305	<0.0001
**Ba/F3**	**Mean 1**	**Mean 2**	**s.d 1**	**s.d 2**	**Mean Difference**	**95% CI of difference**	**Adjusted P Value**
Jurkat vs. V658F	0.9944	0.5867	0.0124	0.0075	0.4077	0.3875 to 0.4279	<0.0001
Jurkat vs. Q988P	0.9944	0.6375	0.0124	0.0085	0.3569	0.3367 to 0.3772	<0.0001
Jurkat vs. N642H	0.9944	0.5142	0.0124	0.0037	0.4802	0.4599 to 0.5004	<0.0001
**Figure 3E**	**Two-way ANOVA; Dunnett's multiple comparisons test**						
**M07e**	**Mean 1**	**Mean 2**	**s.d 1**	**s.d 2**	**Mean Difference**	**95% CI of difference**	**Adjusted P Value**

**G1 phase**							
Jurkat vs. V658F	1.001	1.648	0.0277	0.0652	-0.6462	-0.7237 to -0.5687	<0.0001
Jurkat vs. Q988P	1.001	1.609	0.0277	0.0310	-0.6073	-0.6848 to -0.5298	<0.0001
Jurkat vs. N642H	1.001	1.514	0.0277	0.0351	-0.5124	-0.5899 to -0.4349	<0.0001
**S phase**							
Jurkat vs. V658F	1.013	0.6492	0.0260	0.0323	0.3642	0.2867 to 0.4417	<0.0001
Jurkat vs. Q988P	1.013	0.6843	0.0260	0.0156	0.3291	0.2515 to 0.4066	<0.0001
Jurkat vs. N642H	1.013	0.5261	0.0260	0.0355	0.4873	0.4098 to 0.5648	<0.0001
**Ba/F3**	**Mean 1**	**Mean 2**	**s.d 1**	**s.d 2**	**Mean Difference**	**95% CI of difference**	**Adjusted P Value**
**G1 phase**							
Jurkat vs. V658F	1.001	1.543	0.0277	0.1332	-0.5416	-0.6697 to -0.4134	<0.0001
Jurkat vs. Q988P	1.001	1.455	0.0277	0.0475	-0.4539	-0.5821 to -0.3258	<0.0001
Jurkat vs. N642H	1.001	1.523	0.0277	0.0310	-0.5215	-0.6497 to -0.3933	<0.0001
**S phase**							
Jurkat vs. V658F	1.013	0.6828	0.0260	0.0650	0.3305	0.2024 to 0.4587	<0.0001
Jurkat vs. Q988P	1.013	0.7374	0.0260	0.0146	0.276	0.1478 to 0.4042	0.0003
Jurkat vs. N642H	1.013	0.5428	0.0260	0.0364	0.4705	0.3424 to 0.5987	<0.0001
**Figure 5D**	**Two-way ANOVA; Dunnett's multiple comparisons test**						
**M07e cell growth**	**Mean 1**	**Mean 2**	**s.d 1**	**s.d 2**	**Mean Difference**	**95% CI of difference**	**Adjusted P Value**
**Q988P**							
0 vs. 10	1.0000	0.4599	0.0499	0.0657	0.5401	0.3638 to 0.7164	<0.0001
0 vs. 50	1.0000	0.4179	0.0499	0.0484	0.5821	0.4058 to 0.7584	<0.0001
0 vs. 100	1.0000	0.3942	0.0499	0.1031	0.6058	0.4295 to 0.7821	<0.0001
**V658F**							
0 vs. 10	1.0000	0.4517	0.0594	0.0548	0.5483	0.3721 to 0,7246	<0.0001
0 vs. 50	1.0000	0.4374	0.0594	0.0190	0.5626	0.3863 to 0.7389	<0.0001
0 vs. 100	1.0000	0.4849	0.0594	0.0743	0.5151	0.3388 to 0.6913	<0.0001

**N642H**							
0 vs. 10	1.0000	0.5786	0.1930	0.0786	0.4214	0.2451 to 0.5977	<0.0001
0 vs. 50	1.0000	0.4902	0.1930	0.0428	0.5098	0.3335 to 0.6860	<0.0001
0 vs. 100	1.0000	0.4995	0.1930	0.0881	0.5005	0.3242 to 0.6768	<0.0001
**M07e viability**	**Mean 1**	**Mean 2**	**s.d 1**	**s.d 2**	**Mean Difference**	**95% CI of difference**	**Adjusted P Value**
**Q988P**							
0 vs. 10	1.0000	0.8613	0.0276	0.0540	0.1387	0.04828 to 0.2291	0.0027
0 vs. 50	1.0000	0.7847	0.0276	0.0334	0.2153	0.1249 to 0.3057	<0.0001
0 vs. 100	1.0000	0.8212	0.0276	0.0666	0.1788	0.08843 to 0.2692	0.0002
**V658F**							
0 vs. 10	1.0000	0.9016	0.0123	0.0742	0.0984	0.008018 to 0.1888	0.0315
0 vs. 50	1.0000	0.8291	0.0123	0.0558	0.1709	0.08049 to 0.2613	0.0004
0 vs. 100	1.0000	0.8377	0.0123	0.0371	0.1623	0.07193 to 0.2527	0.0006
**N642H**							
0 vs. 10	1.0000	0.8521	0.0294	0.0421	0.1479	0.05745 to 0.2383	0.0015
0 vs. 50	1.0000	0.8794	0.0294	0.0576	0.1206	0.03022 to 0.2110	0.0083
0 vs. 100	1.0000	0.8599	0.0294	0.0643	0.1401	0.04967 to 0.2305	0.0025
**Figure S1A**	**Unpaired t test: two-tailed**						
	**Mean 1 ± s.d**	**Mean 2 ± s.d**	**t**	**df**	**Mean Difference (2-1)**	**95% CI of difference**	**P Value**
**MOLT4**							
E.V vs Q988P	1.00 ± 0.0903	3.879 ± 0.7694	6.4380	4	2.8790	1.637 to 4.121	0.0030
**Jurkat**							
E.V vs Q988P	1.025 ± 0.2931	1.796 ± 0.2097	3.7040	4	0.7707	0.1931 to 1.348	0.0208
**HPB-ALL**							
E.V vs Q988P	1.00 ± 0.1842	4.007 ± 0.4767	10.1900	4	3.0070	2,188 to 3,826	0.0005
**Figure S1B**	**One-way ANOVA; Dunnett's multiple comparisons test**						

**M07e cell growth**	**Mean 1**	**Mean 2**	**s.d 1**	**s.d 2**	**Mean Difference**	**95% CI of difference**	**Adjusted P Value**
**Q988P**							
0 vs WT	0.2567	0.2200	0.0737	0.0361	0.0367	-0.1878 to 0.2612	0.8577
0 vs Q988P	0.2567	2.5730	0.0737	0.1447	-2.3170	-2.541 to -2.092	<0.0001
**V658F**							
0 vs WT	0.2500	0.2300	0.0300	0.0500	0.0200	-0.1152 to 0.1552	0.8808
0 vs V658F	0.2500	2.1370	0.0300	0.0814	-1.8870	-2.022 to -1.751	<0.0001
**N642H**							
0 vs WT	0.2733	0.2100	0.0416	0.0361	0.0633	-0.07475 to 0.2014	0.3760
0 vs N642H	0.2733	1.7270	0.0416	0.0862	-1.4530	-1,591 to -1,315	<0.0001
**M07e viability**	**Mean 1**	**Mean 2**	**s.d 1**	**s.d 2**	**Mean Difference**	**95% CI of difference**	**Adjusted P Value**
**Q988P**							
0 vs WT	25.6700	22.0000	7.2342	7.2111	3.6670	-10.72 to 18.06	0.7023
0 vs Q988P	25.6700	87.3300	7.2342	3.0551	-61.6700	-76.06 to -47.28	<0.0001
**V658F**							
0 vs WT	23.6700	22.3300	7.3711	2.5166	1.3330	-9.714 to 12.38	0.9180
0 vs V658F	23.6700	89.6700	7.3711	2.5166	-66.0000	-77.05 to -54.95	<0.0001
**N642H**							
0 vs WT	34.0000	30.3300	12.000<br>0	4.7258	3.6670	-15.07 to 22.40	0.8053
0 vs N642H	34.0000	94.6700	12.000<br>0	5.1316	-60.6700	-79.40 to -41.93	0.0002
**Figure S1C**	**One-way ANOVA; Dunnett's multiple comparisons test**						
**Ba/F3 cell growth**	**Mean 1**	**Mean 2**	**s.d 1**	**s.d 2**	**Mean Difference**	**95% CI of difference**	**Adjusted P Value**
**Q988P**							
0 vs WT	0.3833	0.3633	0.1168	0.0777	0.0200	-0.2735 to 0.3135	0.9727
0 vs Q988P	0.3833	5.1230	0.1168	0.1662	-4.7400	-5.034 to -4.446	<0.0001
**V658F**							

0 vs WT	0.4333	0.3467	0.0379	0.0987	0.0867	-0.2202 to 0.3935	0.6533
0 vs V658F	0.4333	4.5870	0.0379	0.2013	-4.1530	-4.460 to -3.846	<0.0001
**N642H**							
0 vs WT	0.4167	0.3933	0.0416	0.1102	0.0233	-0.4827 to 0.5293	0.9873
0 vs N642H	0.4167	3.9900	0.0416	0.3559	-3.5730	-4.079 to -3.067	<0.0001
**Ba/F3 viability**	**Mean 1**	**Mean 2**	**s.d 1**	**s.d 2**	**Mean Difference**	**95% CI of difference**	**Adjusted P Value**
**Q988P**							
0 vs WT	48.0000	44.6700	7.0000	6.0277	3.3330	-9.207 to 15.87	0.6828
0 vs Q988P	48.0000	97.0000	7.0000	1.0000	-49.0000	-61.54 to -36.46	<0.0001
**V658F**							
0 vs WT	43.6700	42.6700	10.116 0	12.662 3	1.0000	-21.14 to 23.14	0.9878
0 vs V658F	43.6700	94.6700	10.116 0	2.5166	-51.0000	-73.14 to -28.86	0.001
**N642H**							
0 vs WT	39.3300	46.6700	8.1445	5.6862	-7.3330	-21.36 to 6.693	0.2995
0 vs N642H	39.3300	93.6700	8.1445	3.0551	-54.3300	-68.36 to -40.31	<0.0001
**Figure S2A**	**One-way ANOVA; Dunnett's multiple comparisons test**						
**Ba/F3**	**Mean 1**	**Mean 2**	**s.d 1**	**s.d 2**	**Mean Difference**	**95% CI of difference**	**Adjusted P Value**
**Q988P**							
0 vs WT	1.0000	1.1830	0.0346	0.0493	-0.1833	-0.5374 to 0.1708	0.3051
0 vs Q988P	1.0000	11.9900	0.0346	0.2553	-10.9900	-11.34 to -10.64	<0.0001
**V658F**							
0 vs WT	1.0000	1.1100	0.0200	0.0361	-0.1100	-0.2537 to 0.03368	0.1205
0 vs V658F	1.0000	12.3500	0.0200	0.0981	-11.3500	-11.50 to -11.21	<0.0001
**N642H**							
0 vs WT	1.0070	1.0670	0.0153	0.0802	-0.0600	-0.5054 to 0.3854	0.8995
0 vs N642H	1.0070	8.1330	0.0153	0.3197	-7.1270	-7.572 to -6.681	<0.0001

Figure S4C	Two-way ANOVA; Dunnett's multiple comparisons test						
**Ba/F3 cell growth**	**Mean 1**	**Mean 2**	**s.d 1**	**s.d 2**	**Mean Difference**	**95% CI of difference**	**Adjusted P Value**
**Q988P**							
0 vs. 10	1.0000	0.4912	0.0189	0.0173	0.5088	0.4209 to 0.5967	<0.0001
0 vs. 50	1.0000	0.4714	0.0189	0.0244	0.5286	0.4407 to 0.6165	<0.0001
0 vs. 100	1.0000	0.4581	0.0189	0.0175	0.5419	0.4540 to 0.6298	<0.0001
**V658F**							
0 vs. 10	1.0000	0.4982	0.0548	0.0373	0.5018	0.4139 to 0.5897	<0.0001
0 vs. 50	1.0000	0.4676	0.0548	0.0211	0.5324	0.4445 to 0.6203	<0.0001
0 vs. 100	1.0000	0.4562	0.0548	0.0101	0.5438	0.4559 to 0.6317	<0.0001
**N642H**							
0 vs. 10	1.0000	0.4237	0.0405	0.0720	0.5763	0.4884 to 0,6642	<0.0001
0 vs. 50	1.0000	0.3056	0.0405	0.0606	0.6944	0.6065 to 0.7823	<0.0001
0 vs. 100	1.0000	0.3409	0.0405	0.0631	0.6591	0.5712 to 0.7470	<0.0001
**Ba/F3 viability**	**Mean 1**	**Mean 2**	**s.d 1**	**s.d 2**	**Mean Difference**	**95% CI of difference**	**Adjusted P Value**
**Q988P**							
0 vs. 10	1.0000	0.9948	0.0092	0.0172	0.0052	-0.03377 to 0.04424	0.9725
0 vs. 50	1.0000	0.9885	0.0092	0.0194	0.0115	-0.02754 to 0.05047	0.7936
0 vs. 100	1.0000	0.9880	0.0092	0.0427	0.0120	-0.02697 to 0.05103	0.7708
**V658F**							
0 vs. 10	1.0000	1.0130	0.0252	0.0287	-0.0132	-0.05224 to 0.02576	0.7197
0 vs. 50	1.0000	0.9874	0.0252	0.0134	0.0126	-0.02639 to 0.05161	0.7465
0 vs. 100	1.0000	0.9904	0.0252	0.0518	0.0096	-0.02945 to 0.04856	0.8649
**N642H**							
0 vs. 10	1.0000	0.9636	0.0103	0.0091	0.0364	-0.002612 to 0.07539	0.0702
0 vs. 50	1.0000	0.9722	0.0103	0.0169	0.0278	-0.01118 to 0.06682	0.1971
0 vs. 100	1.0000	0.9816	0.0103	0.0175	0.0184	-0.02060 to 0.05740	0.4969

SUPPLEMENTARY FIGURES AND LEGENDS

Supplementary Figure 1

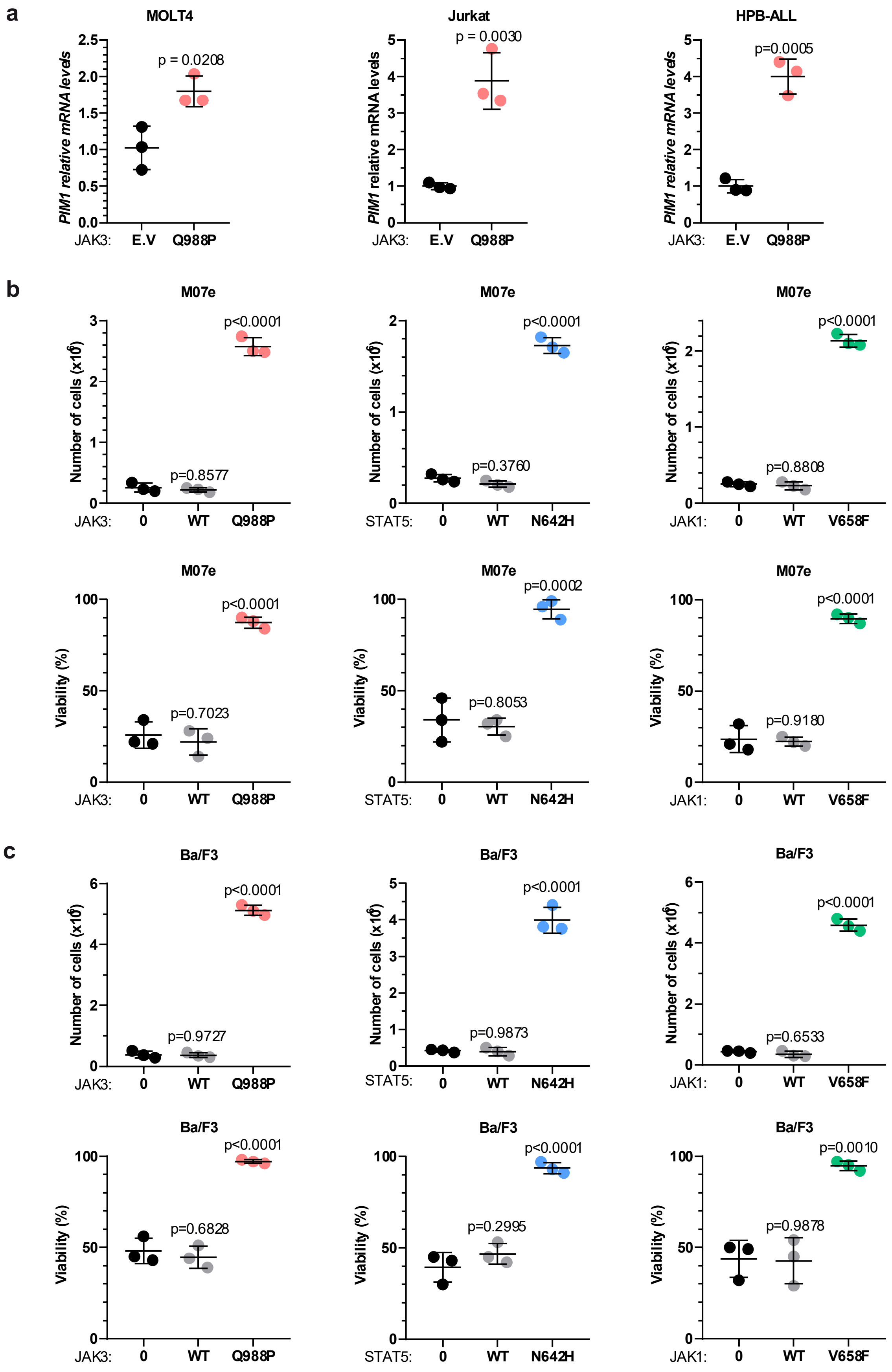

Supplementary Figure 1. (a) mRNA expression levels by RT-qPCR of *PIM1* in MOLT4, Jurkat and HPB-ALL transduced with $JAK3^{Q988P}$ mutation compared with the empty vector (E.V). (b) Cell growth (top) and viability (bottom) assays of M07e cells transduced with $JAK1^{V658F}$, $JAK3^{Q988P}$ or $STAT5^{N642H}$ compared to non-transduced cells (0) and cells transduced with the corresponding wild-type sequence. (c) Cell growth (top) and viability (bottom) assays of Ba/F3 cells transduced with $JAK1^{V658F}$, $JAK3^{Q988P}$ or $STAT5^{N642H}$ compared to non-transduced cells (0) and cells transduced with the corresponding wild-type sequence. The graphics show the mean ± standard deviation (s.d.) after three independent experiments. Statistical comparisons are made against non-transduced cells (0)

Supplementary Figure 2

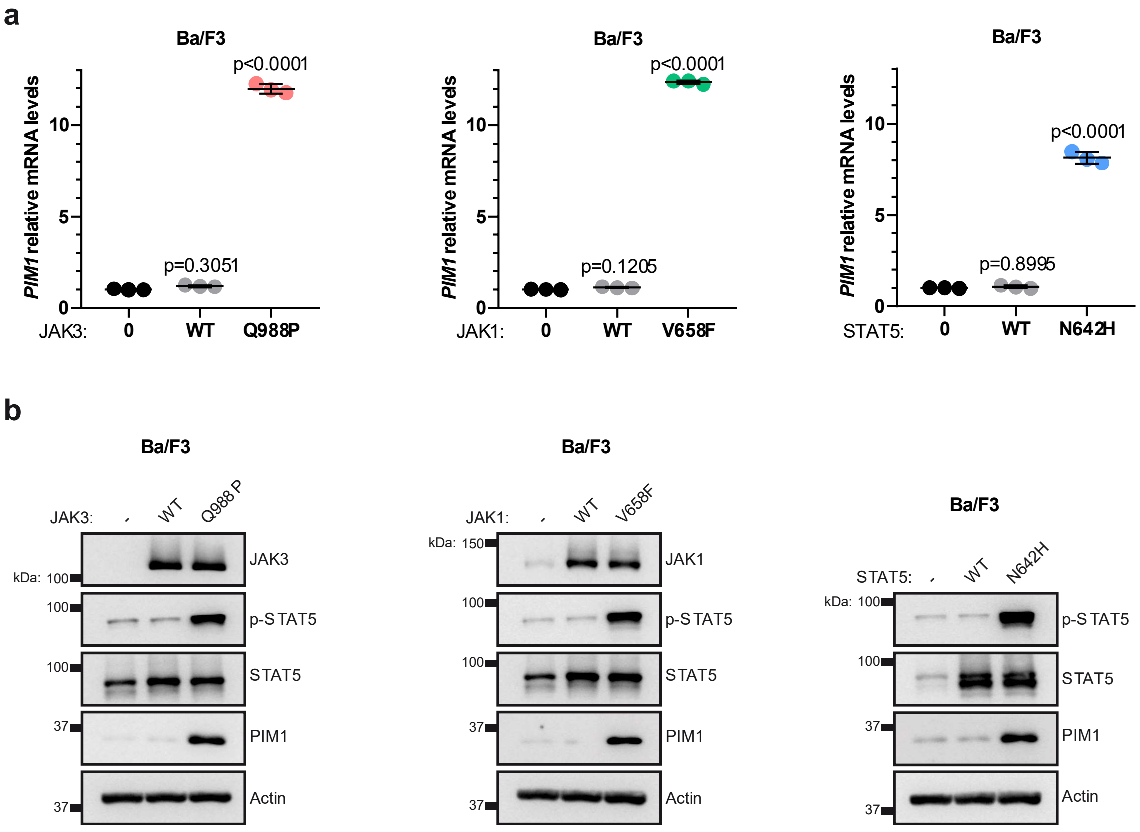

Supplementary Figure 2. (a) mRNA expression levels by RT-qPCR of *PIM1* in Ba/F3 cells transduced with JAK3^{Q988P}, JAK1^{V658F} or STAT5^{N642H} compared to cells transduced with the corresponding wild-type sequence and non-transduced cells (0). Statistical comparisons are made against non-transduced cells (0). (b) Western blot for JAK1, JAK3, p-STAT5, STAT5 and PIM1 in Ba/F3 cells transduced with JAK3^{Q988P}, JAK1^{V658F} or STAT5^{N642H} compared to cells transduced with the corresponding wild-type sequence and non-transduced cells (-). The graphics show the mean ± standard deviation (s.d.) after three independent experiments. All images are representative examples of at least three independent experiments.

Supplementary Figure 3

a **b**

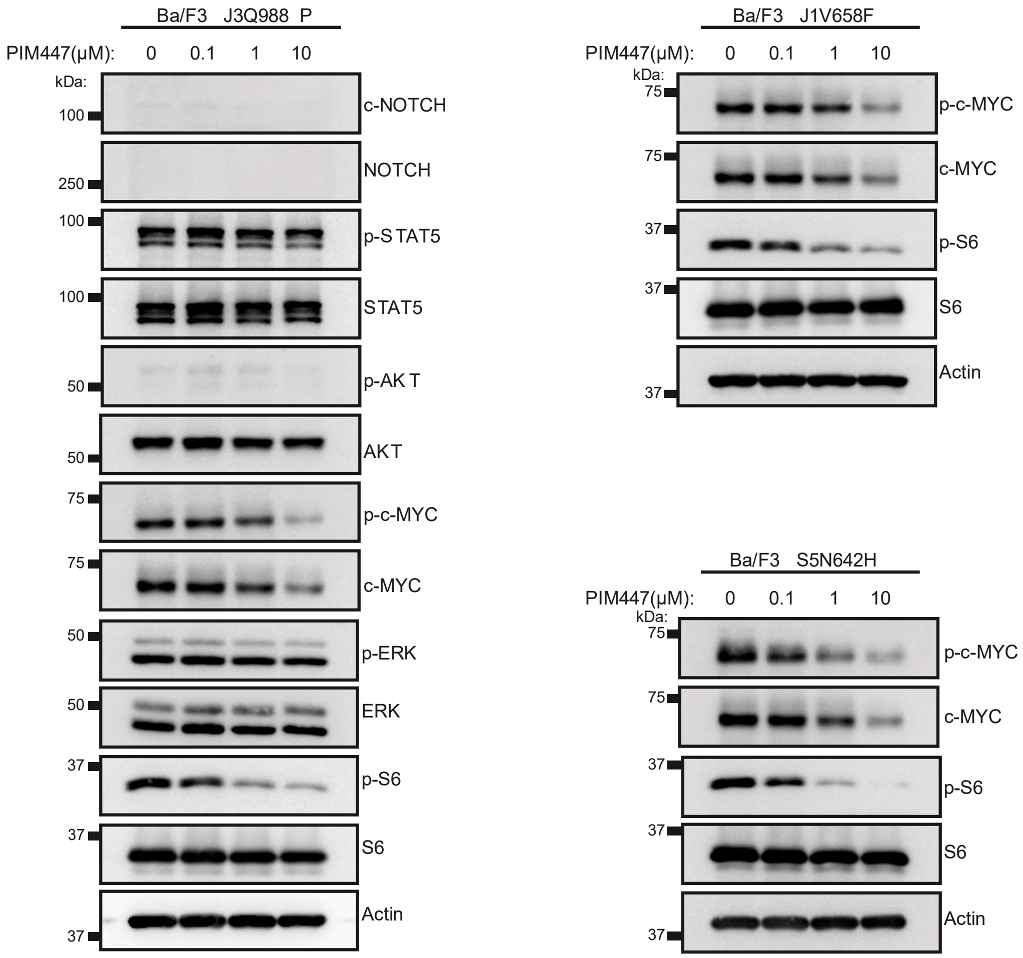

Supplementary Figure 3. (a) Western blot for c-NOTCH1, NOTCH1, p-STAT5, STAT5, p-AKT, AKT, p-c-MYC, c-MYC, p-ERK, ERK, p-S6 and S6 in Ba/F3 cells transduced with $JAK3^{Q988P}$ untreated or treated with PIM447 (0.1µM, 1µM or 10µM). (b) Western blot for p-c-MYC, c-MYC, p-S6 and S6 in Ba/F3 cells transduced with $JAK1^{V658F}$ or $STAT5^{N642H}$ untreated or treated with PIM447 (0.1µM, 1µM or 10µM). All images are representative examples of at least three independent experiments.

Supplementary Figure 4

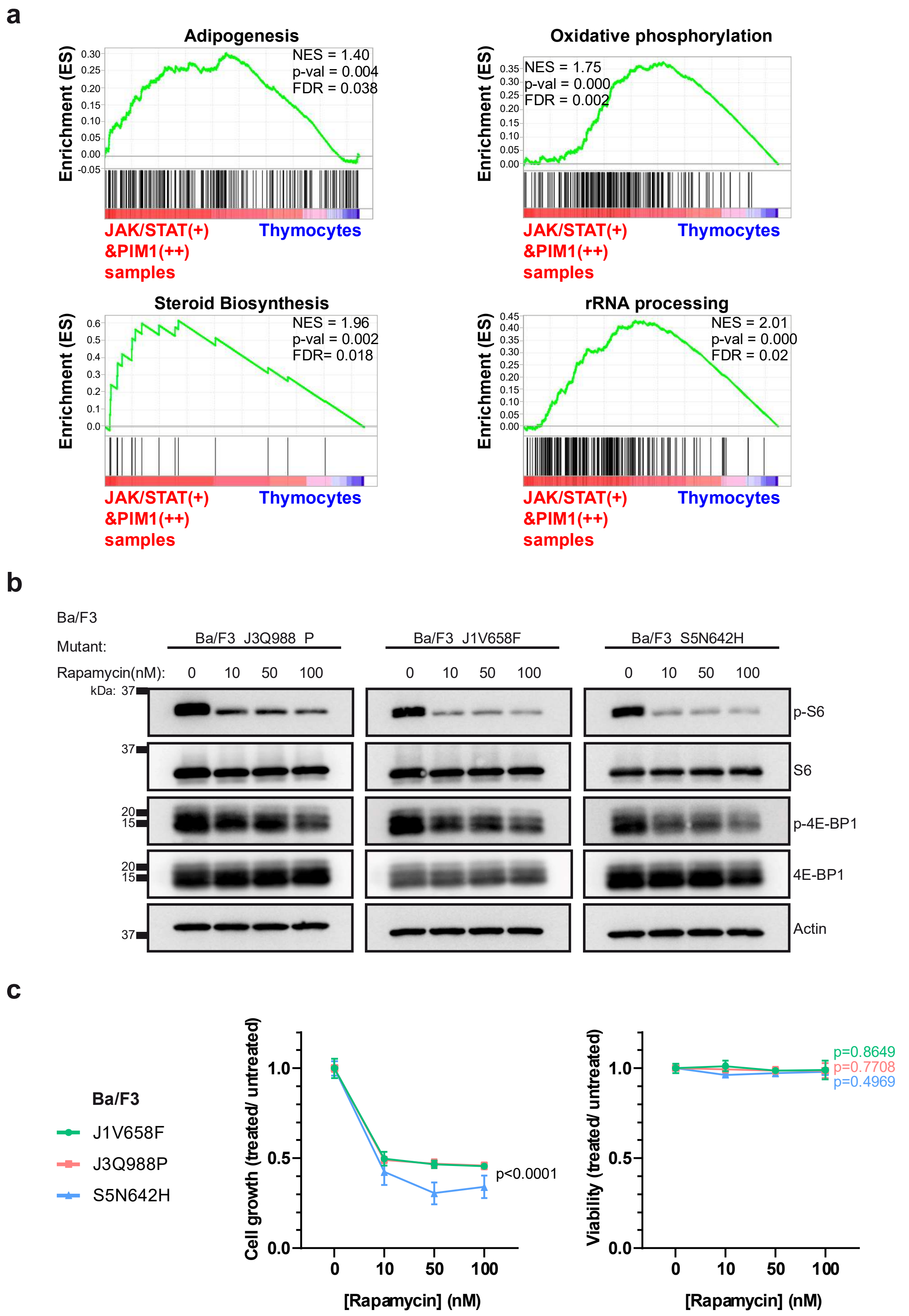

Supplementary Figure 4. (a) GSEA-plots for adipogenesis, oxidative phosphorylation, steroid biosynthesis and rRNA processing in control thymocytes (referred as thymocytes) and T-ALL/LBL samples with JAK/STAT pathway mutations and a twofold increase in *PIM1* expression (referred

as JAK/STAT (+) &PIM1(++) samples). We selected human postnatal thymocytes as the most suitable control since T-ALL/LBL samples without mutations in the JAK/STAT pathway may present other molecular alterations that are susceptible of increasing the GSEA-signatures analyzed. (b) Western blot for p-S6, S6, p-4E-BP1 and 4E-BP1 in Ba/F3 cells transduced with $JAK3^{Q988P}$, $JAK1^{V658F}$ or $STAT5^{N642H}$ and treated with rapamycin (10nM, 50nM or 100nM) or left untreated. (c) Cell growth (left) and viability (right) assays of Ba/F3 cells transduced with $JAK3^{Q988P}$, $JAK1^{V658F}$ or $STAT5^{N642H}$, treated with rapamycin (10nM, 50nM or 100nM) and referred to untreated cells. The graphics show the mean ± standard deviation (s.d.) after three independent experiments. Statistical comparisons are made against untreated cells. All images are representative examples of at least three independent experiments.

Supplementary Figure 5

a Figure 2d (left) — Figure 2d (middle) — Figure 2d (right)

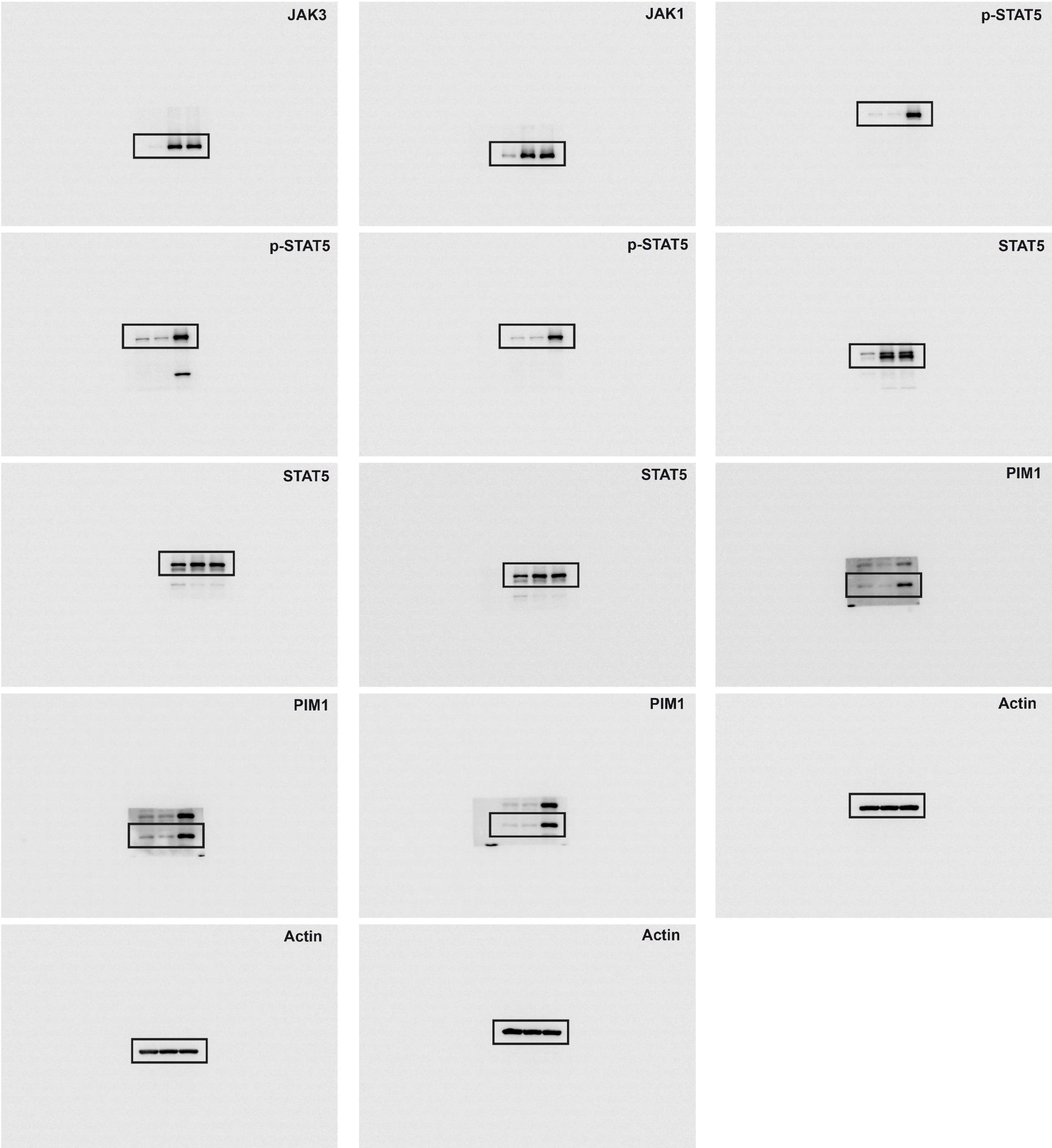

b Figure 2f

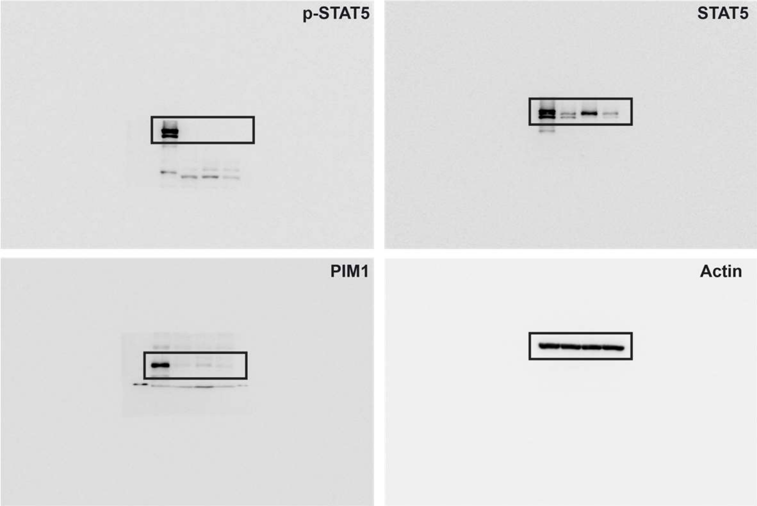

c Figure 2h

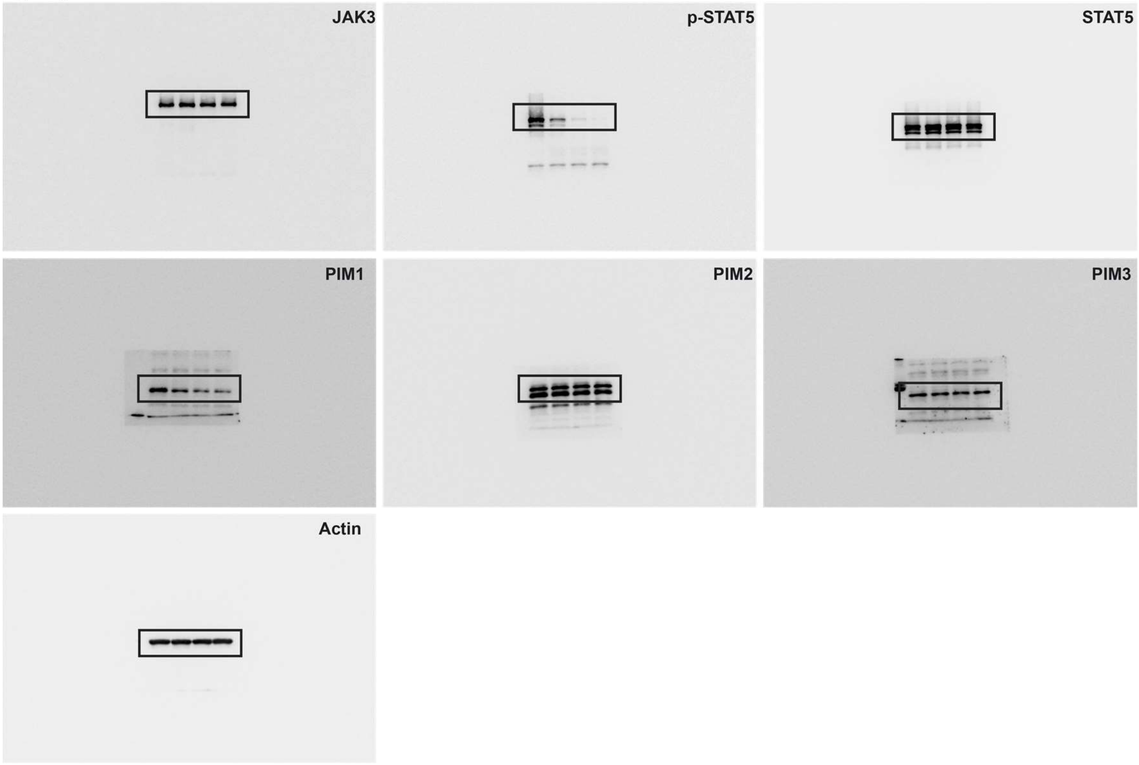

d Figure 3g (left)

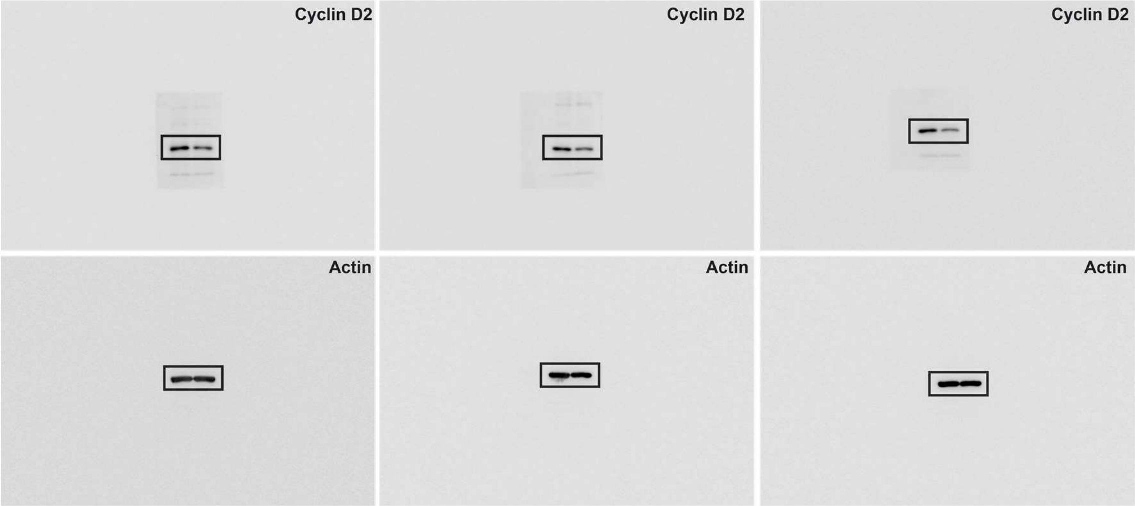

Figure 3g (right)

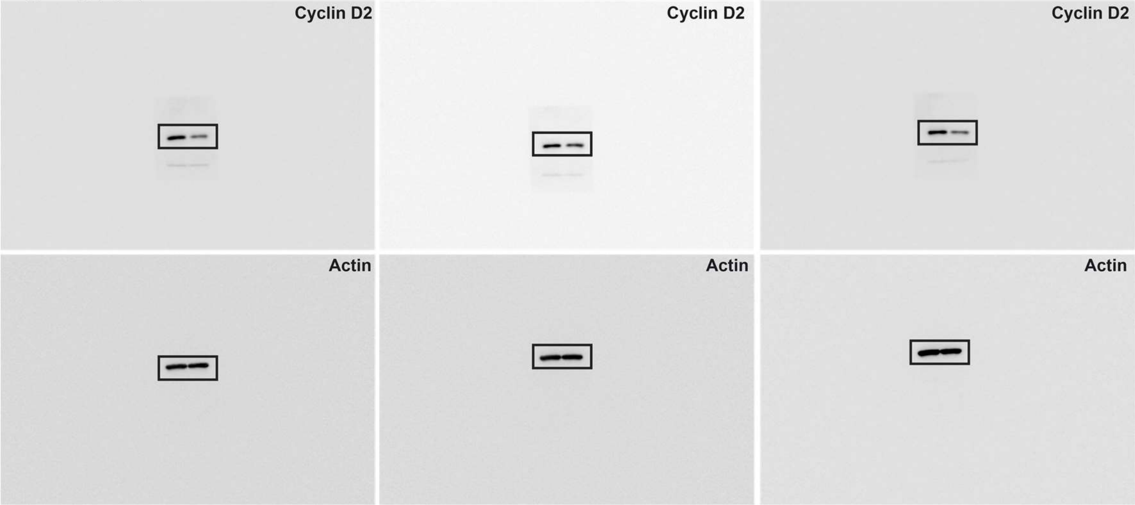

e Figure 4c

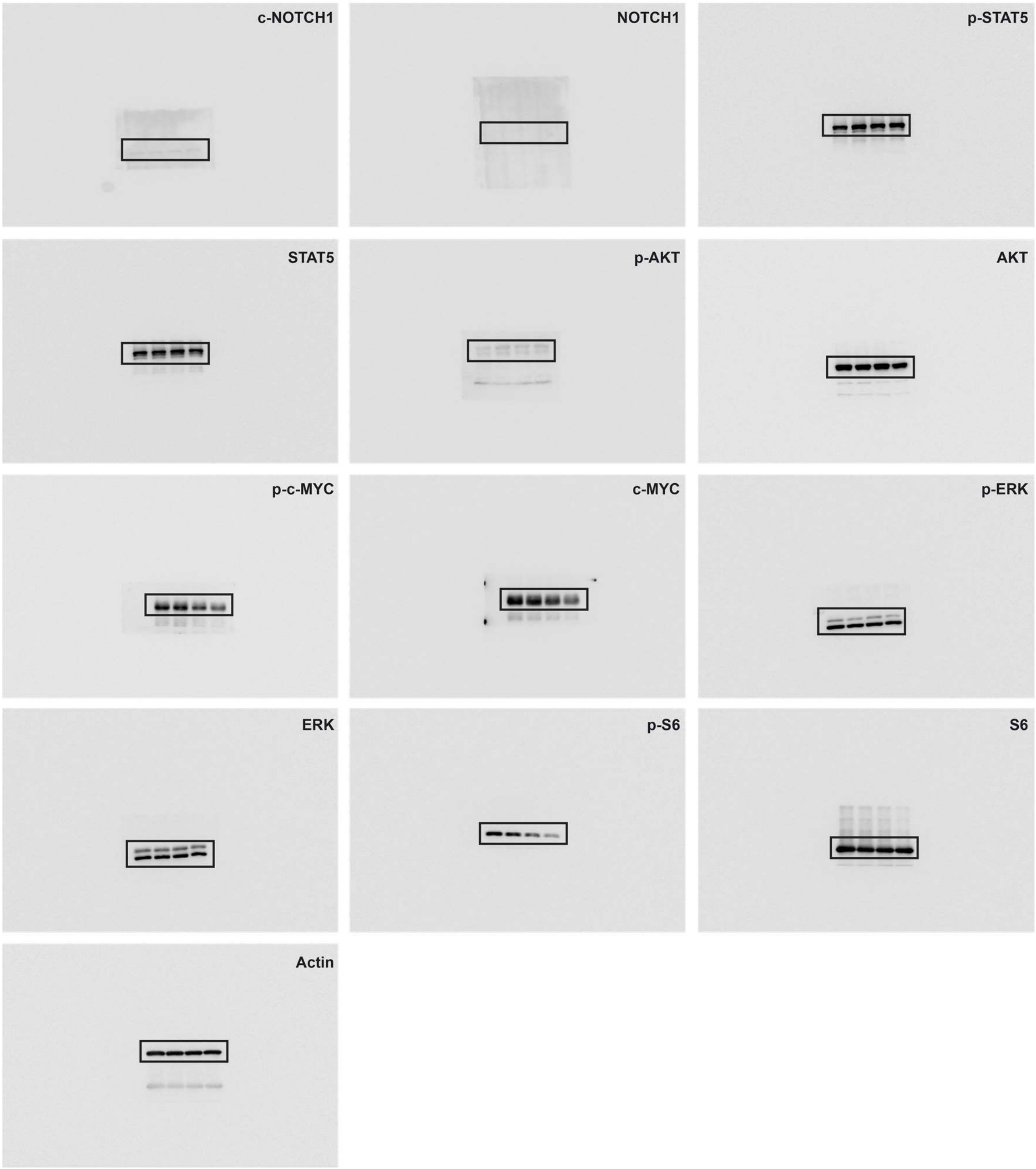
c-NOTCH1
NOTCH1
p-STAT5
STAT5
p-AKT
AKT
p-c-MYC
c-MYC
p-ERK
ERK
p-S6
S6
Actin

f

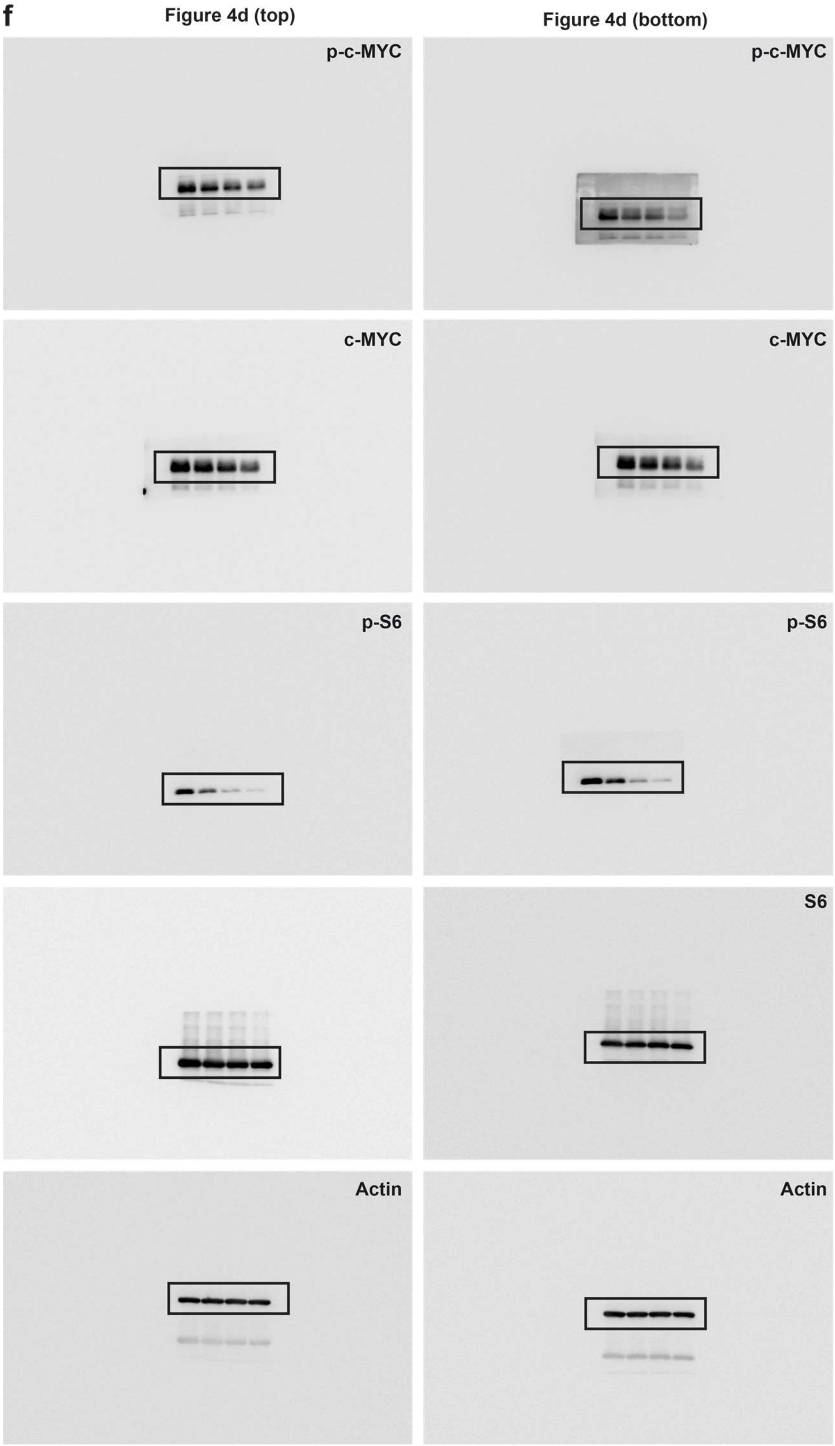
Figure 4d (top)
Figure 4d (bottom)
p-c-MYC
p-c-MYC
c-MYC
c-MYC
p-S6
p-S6
S6
Actin
Actin

g Figure 4e

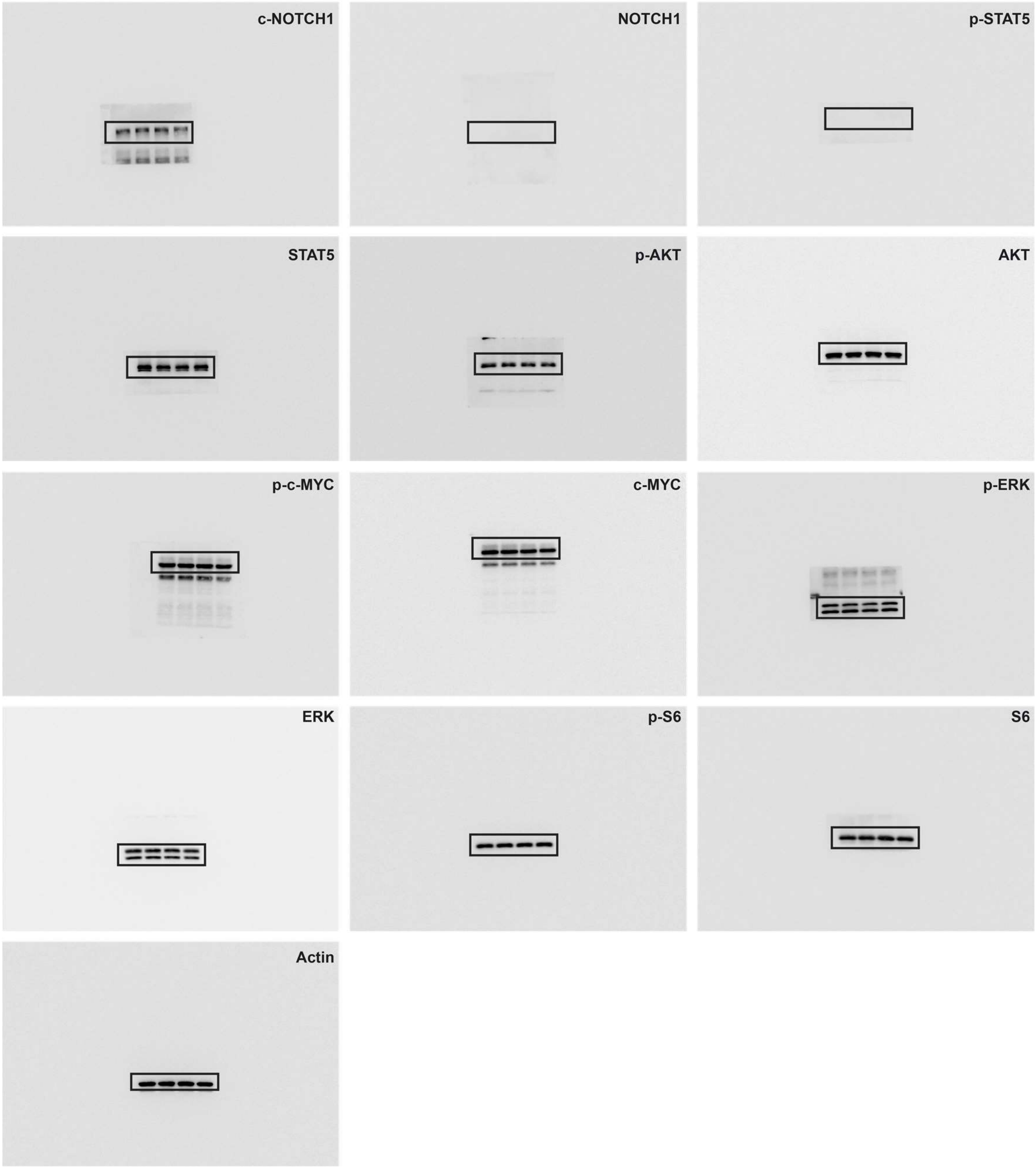
c-NOTCH1
NOTCH1
p-STAT5
STAT5
p-AKT
AKT
p-c-MYC
c-MYC
p-ERK
ERK
p-S6
S6
Actin

h

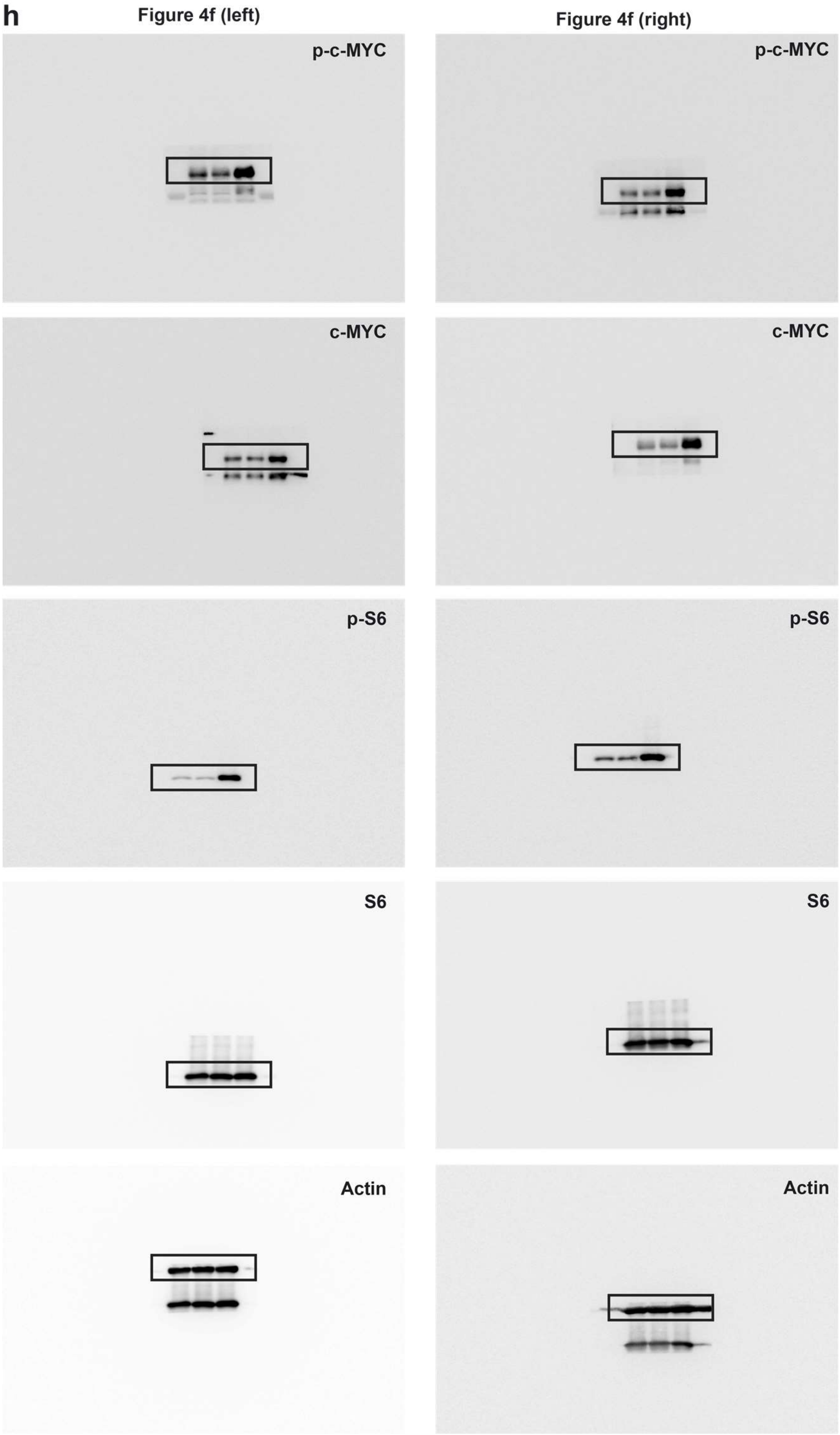
Figure 4f (left)
Figure 4f (right)
p-c-MYC
p-c-MYC
c-MYC
c-MYC
p-S6
p-S6
S6
S6
Actin
Actin

i Figure 5c

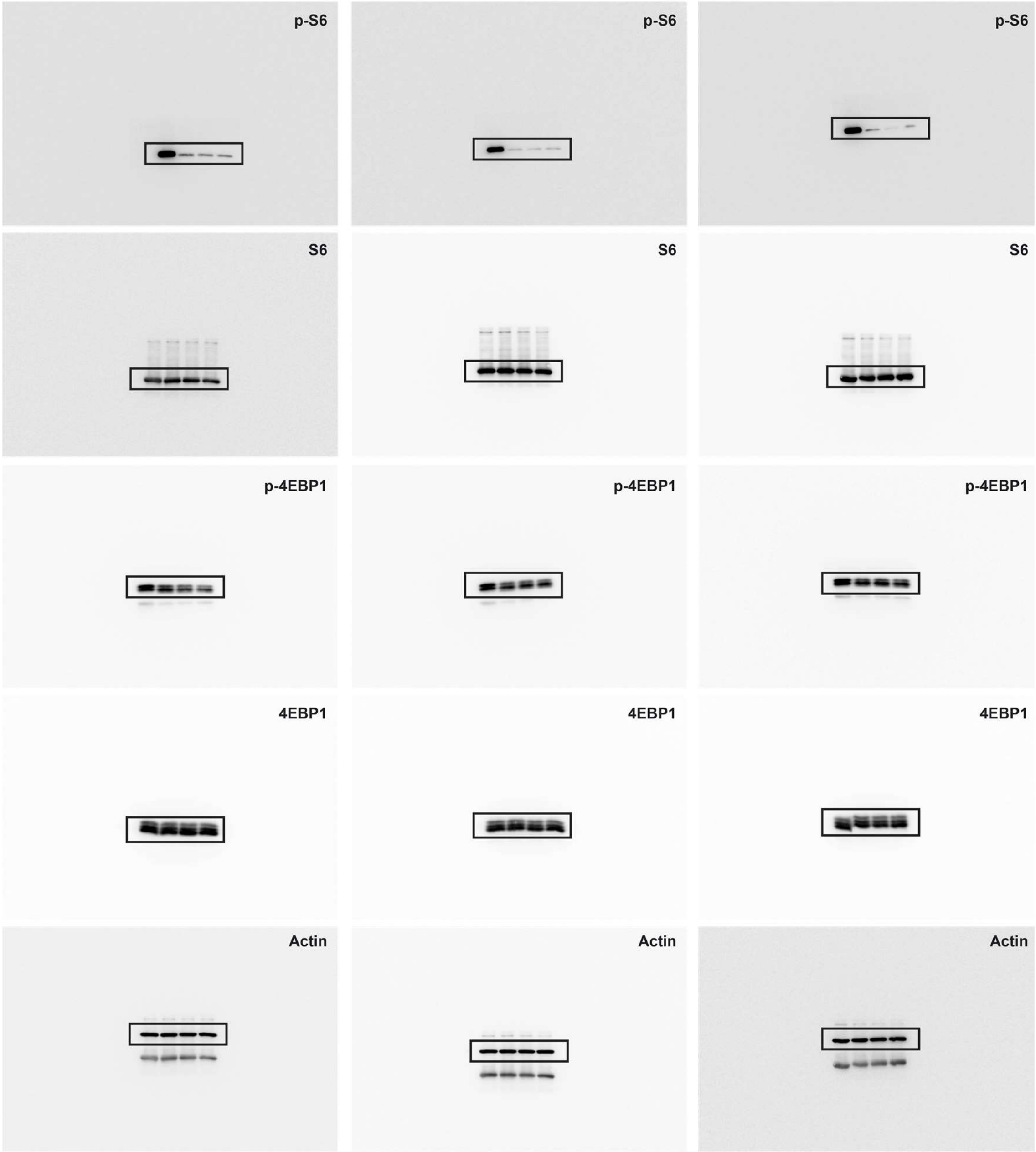
p-S6
p-S6
p-S6
S6
S6
S6
p-4EBP1
p-4EBP1
p-4EBP1
4EBP1
4EBP1
4EBP1
Actin
Actin
Actin

j

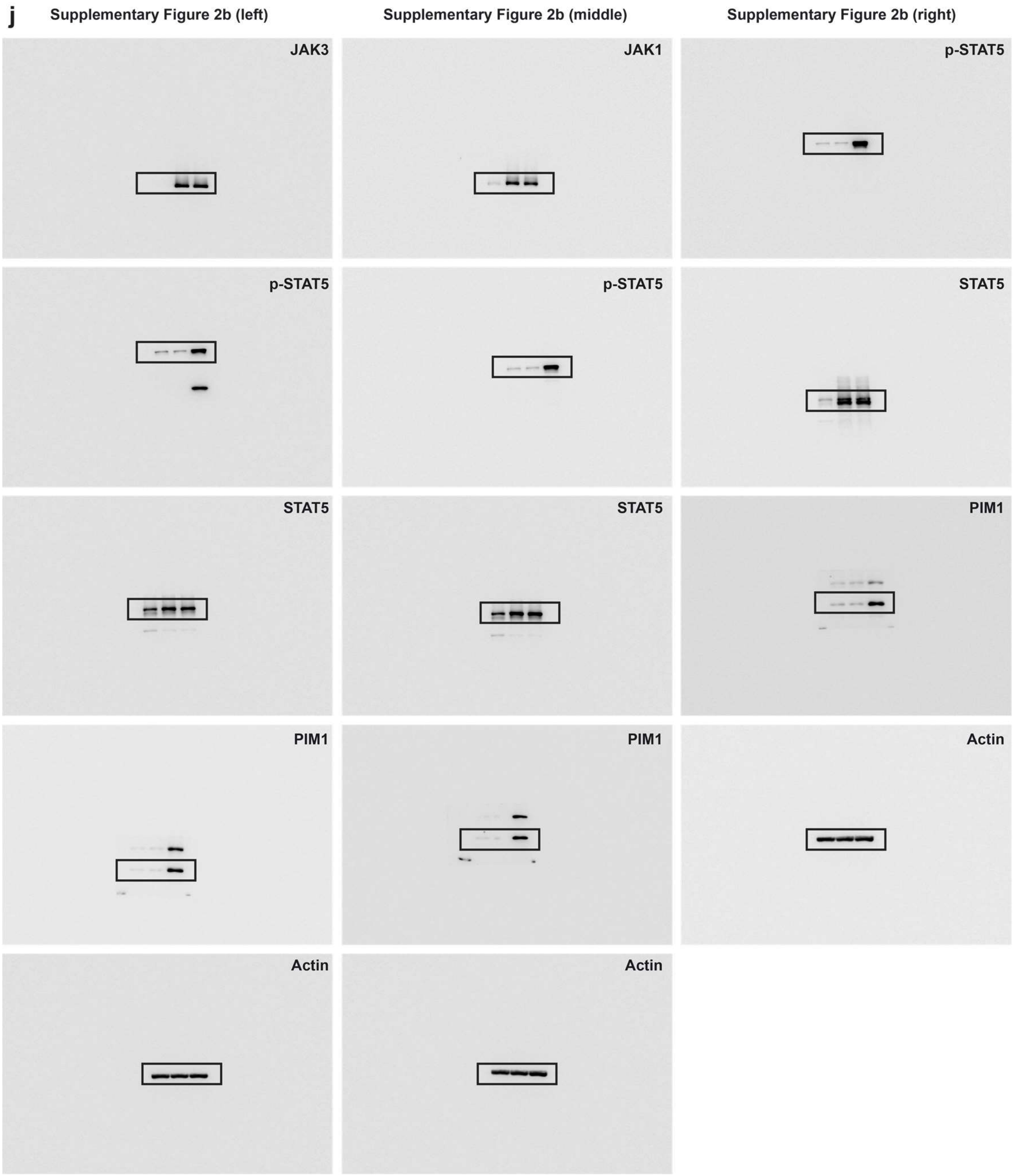
Supplementary Figure 2b (left)
Supplementary Figure 2b (middle)
Supplementary Figure 2b (right)
JAK3
JAK1
p-STAT5
p-STAT5
p-STAT5
STAT5
STAT5
STAT5
PIM1
PIM1
PIM1
Actin
Actin
Actin

k Supplementary Figure 3a

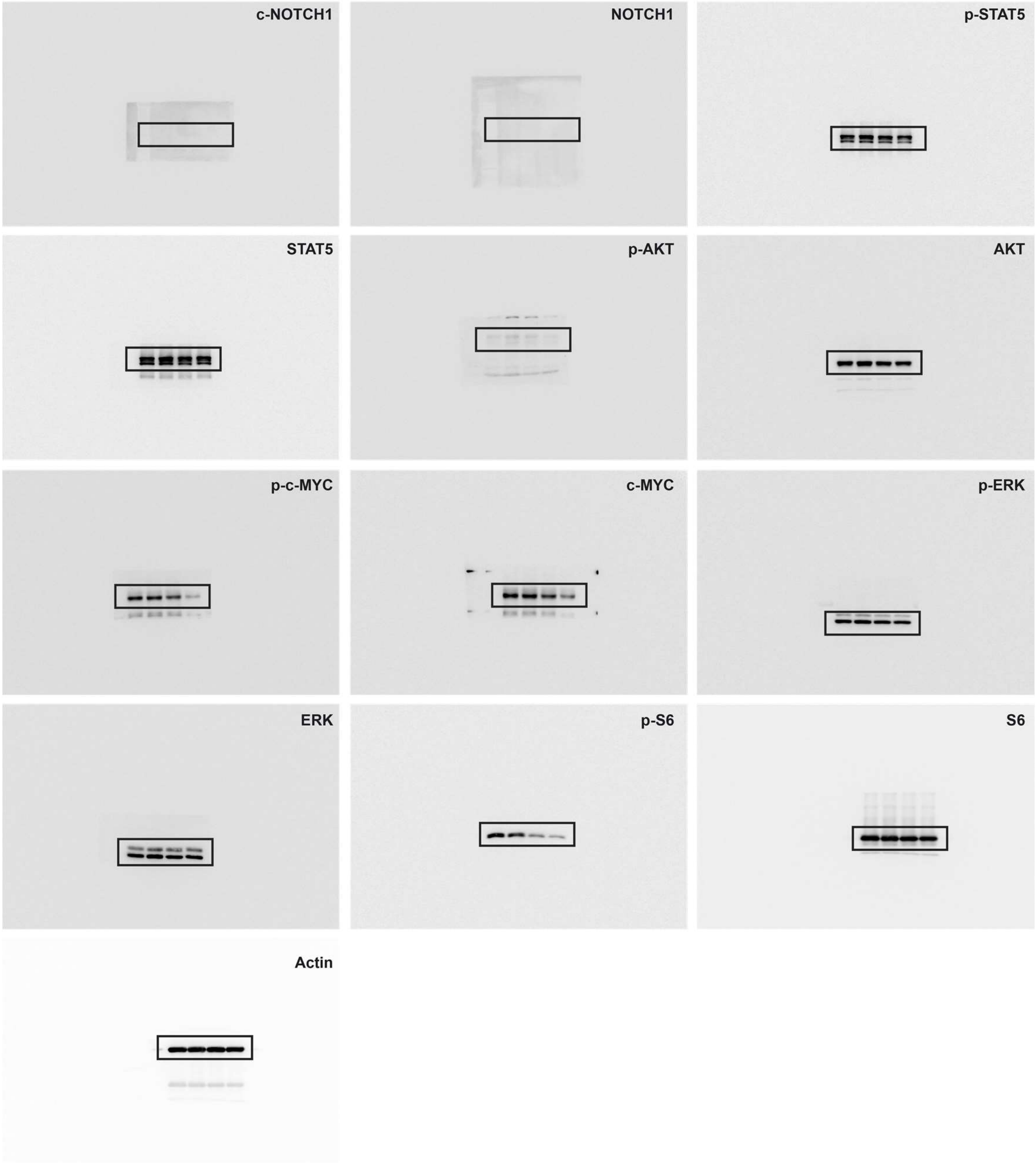

l Supplementary Figure 3b (top) | Supplementary Figure 3b (bottom)

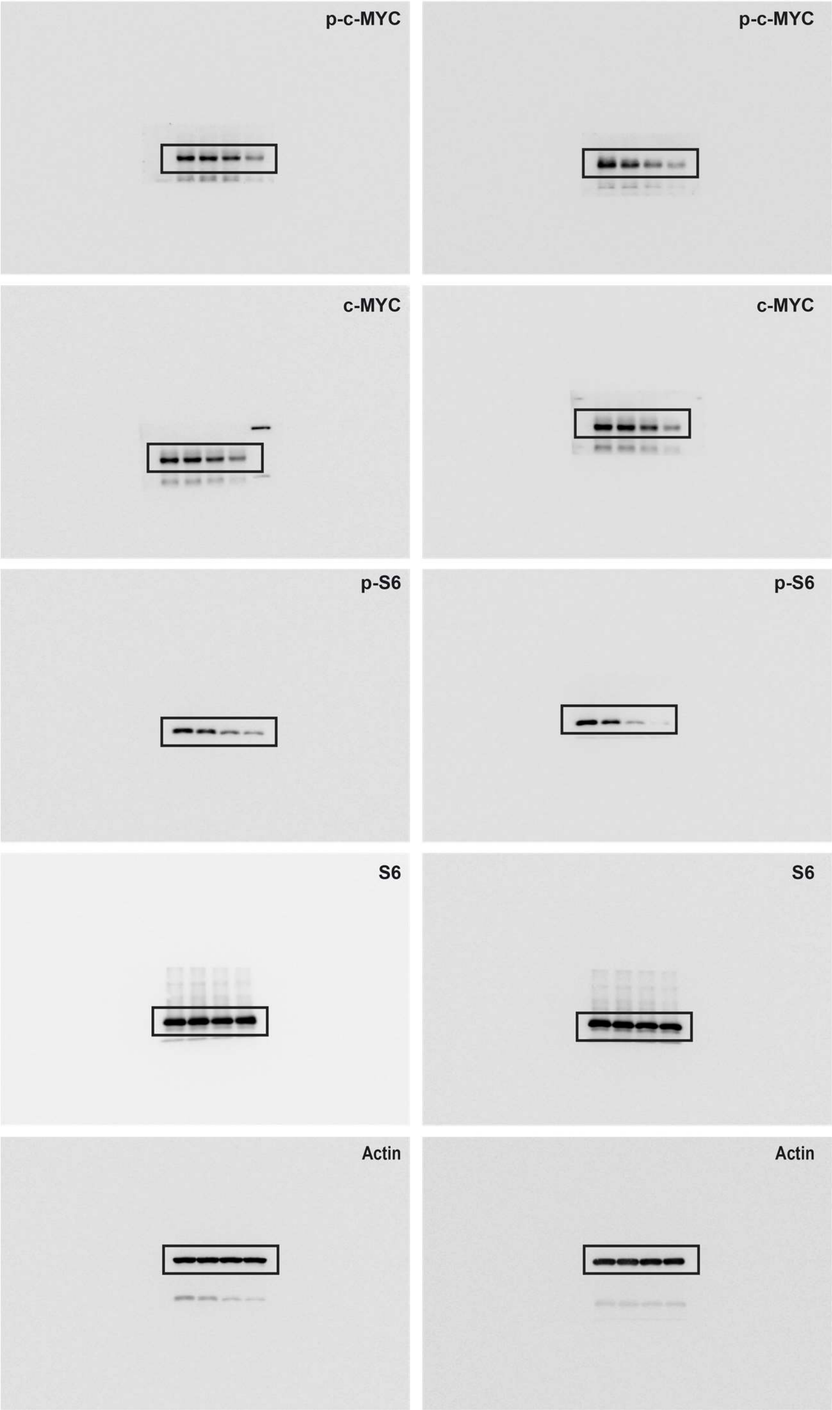

m Supplementary Figure 4b

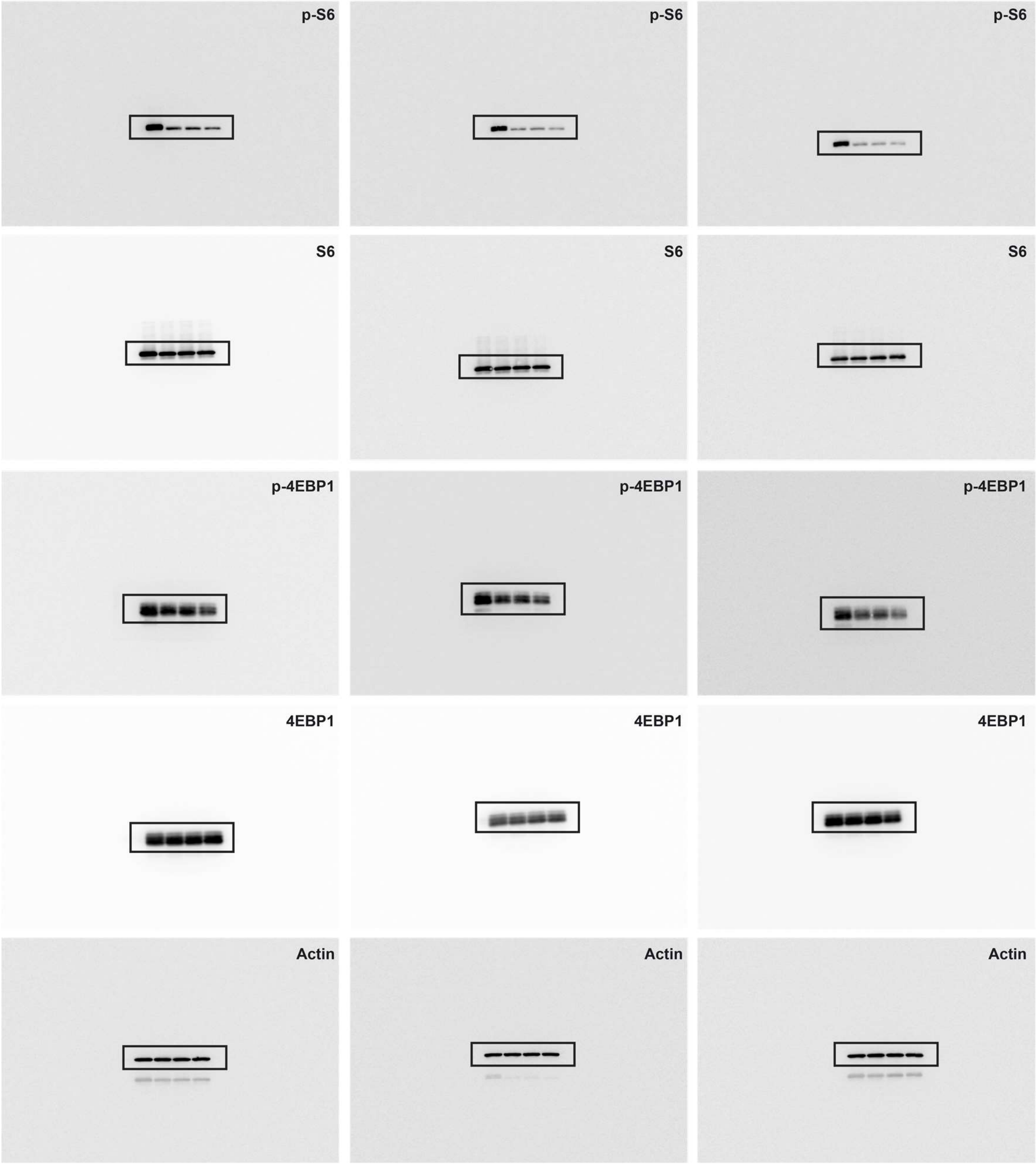

Supplementary Figure 5. Uncropped, unmodified images of every blot included in the figures are shown. (a) Figure 2d, (b) Figure 2f, (c) Figure 2h, (d) Figure 3g, (e) Figure 4c, (f) Figure 4d, (g) Figure 4e, (h) Figure 4f, (i) Figure 5c, (j) Supplementary Figure 2b, (k) Supplementary Figure 3a, (l) Supplementary Figure 3b and (m) Supplementary Figure 4b.

Supplementary Figure 6

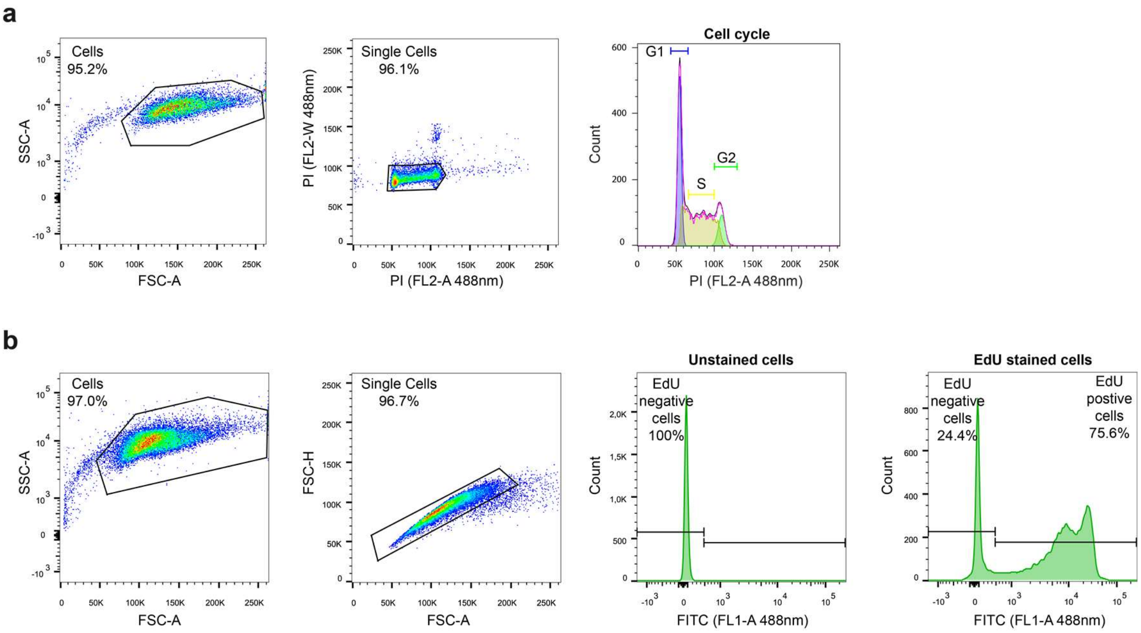

Supplementary Figure 6. Gating strategy used for flow cytometry assays. (a) For cell cycle assay, alive cells were gated on the SSC-A/FSC-A plot and single cells were gated on the FL2-W and FL2-A plot. Cells were dyed with propidium iodide (PI) flurochrome, which is excited at 488nm and detected in the 582/15 filter (FL2). Cell cycle phases were determined using Watson Pragmatic Watson pragmatic fitting algorithm from FlowJo v10. (b) For EdU assay, alive cells were gated on the SSC-A/FSC-A plot and single cells were gated on the FSC-H and FSC-A plot. EdU stained cells were gated according to the unstained samples. The fluorochrome used for EdU experiments was FITC, which is excited at 488nm and detected in the 530/30 filter (FL1).
